# Identification of novel HLA class I-restricted hepatitis B virus peptides and their modulation by peptide editor TAPBPR

**DOI:** 10.3389/fimmu.2026.1892820

**Published:** 2026-07-31

**Authors:** Ricky Sinharay, Dominic S. Nolan, Jens Bauer, Andreas Neerincx, Juliane S. Walz, William Gelson, Arwen F. Altenburg, Louise H. Boyle

**Affiliations:** 1 Department of Pathology, University of Cambridge, Cambridge, United Kingdom; 2 Cambridge Liver Unit, Cambridge University Hospitals National Health Service (NHS) Foundation Trust, Cambridge, United Kingdom; 3 Department of Peptide-based Immunotherapy, Institute of Immunology, University and University Hospital, Tübingen, Germany; 4 Cluster of Excellence iFIT (EXC2180) “Image-Guided and Functionally Instructed Tumor Therapies”, University of Tübingen, Tubingen, Germany; 5 German Cancer Consortium Deutsches Konsortium für Translationale Krebsforschung (DKTK), Partner Site Tübingen, a Partnership Between Deutsches Krebsforschungszentrum (DKFZ) and University Hospital Tübingen, Tubingen, Germany; 6 Clinical Collaboration Unit Translational Immunology, Department of Internal Medicine, University Hospital Tübingen, Tübingen, Germany

**Keywords:** antigen processing & presentation, hepatitis B virus (HBV), HLA class I, immunopeptidomics, MHC-I, peptide discovery, TAPBPR

## Abstract

**Introduction:**

Hepatitis B virus (HBV) is the leading cause of hepatocellular carcinoma and infections remain a substantial global health problem for which no curative therapy exists. As cytotoxic CD8^+^ T lymphocytes are the principal effectors of natural HBV resolution, there is considerable interest in identification of HBV epitopes that are presented for immune recognition. Current knowledge of HBV-derived peptide presentation to CD8^+^ T cells is largely dominated human leukocyte antigen (HLA)-A*02:01. Our aim was to characterize novel HBV-derived peptides presented on HLA-B and -C molecules, particularly allotypes that are prevalent in regions with high HBV infection rates.

**Methods:**

To discover new HBV-derived peptides, immunopeptidomic analysis was performed on cells expressing single HLA class I molecules transduced to express individual HBV proteins. Peptide binding assays were used to validate HLA binding of selected peptides. Additionally, we used immunopeptidomic analysis to determine the effect of the recently discovered major histocompatibility complex class I (MHC-I) peptide editor TAPBPR on presentation of HBV-derived peptides. The results were validated using TAPBPR peptide-exchange assays.

**Results:**

We identified 28 novel HBV-derived peptides presented on HLA class I. Importantly, 22 of the newly discovered HBV-derived peptides were identified on cells expressing HLA-B*08:01, -B*15:03, -B*35:01, -C*06:02 and -C*12:03.

**Discussion:**

This work presents a substantial expansion of the HLA-B and HLA-C-restricted HBV-derived peptides described in the Immune Epitope Database (IEDB). Additionally, TAPBPR^KO^ HeLa cells presented four additional and novel HBV-derived peptides, representing the first indication that TAPBPR shapes not only the cellular but also the viral peptide repertoire presented on MHC-I molecules.

## 1 Introduction

Hepatitis B virus (HBV) infection is a major global health challenge, with an estimated 294 million people living with chronic infection worldwide and over 2,000 deaths each day attributable to HBV-related disease (1). Despite the World Health Organization’s (WHO) target to eliminate HBV as a public health threat by 2030, mortality continues to rise because of cirrhosis and hepatocellular carcinoma (HCC). HCC is now the sixth most common cancer and the third leading cause of cancer-related death globally, with its projected incidence set to increase substantially over coming decades (2, 3). The introduction of an effective preventive vaccine based on recombinant HBV surface antigen (HBsAg) has led to a reduction in global HBV infections (4, 5). However, the impact on rates of advanced liver disease or HCC will not be observed for at least two decades, given the long latency between infection and end-stage complications (6).

HBV is an enveloped, partially double-stranded DNA virus that infects hepatocytes. Transmission is predominantly vertical, with mother-to-child exposure at birth conferring a high risk of chronic disease. While exposure as an adult typically results in self-limiting disease with low morbidity, infection acquired in infancy or early childhood drives prolonged necroinflammatory liver injury. This substantially increases lifetime risk of liver cirrhosis and HCC (7). HBV incidence and prevalence are highest in WHO African, South-East Asian and Western Pacific regions (4, 5, 8–10). While HBV does not display strong long-term mutational variation, there are nine HBV genotypes that differ in geographical distribution, transmission dynamics, treatment response, disease progression and HCC risk (11).

HBV is directly oncogenic through integration of viral DNA into the host hepatocyte genome, leading to the promotion of hepatocyte clonal expansion. Notably, this process occurs in all phases of chronic HBV infection acquired in infancy or childhood, irrespective of inflammatory activity or fibrosis stage (12). Current standard-of-care treatment with nucleos(t)ide analogues (NUCs) effectively suppresses viral replication and mitigates, but does not eliminate, the risk of HCC (13). Immunotherapeutic strategies evaluated to date have failed to achieve the durable long term immune control required for curative therapy, defined as sustained HBsAg loss following a finite course of treatment (14).

CD8^+^ T cells are the main effector cells in the natural resolution of infection and are therefore of interest for novel treatment strategies (15, 16). The CD8^+^ T cell response is polyclonal and directed against the viral capsid-forming core (HBcAg), pre-core e antigen (HBeAg), HBsAg (S, PreS1, PreS2), viral polymerase (Pol) and the non-structural x antigen (HBxAg) (17–25). Effector CD8^+^ T cells lyse infected target cells and can abolish HBV replication by non-cytopathic mechanisms through the release of cytokines (15, 26). HBV persistence is associated with impaired CD8^+^ T cell responses (17–19). NUC treatment results in limited restoration of HBV-specific T cell function and may be more effective in combination with immunomodulatory therapies that further enhance T cell activity (14, 27). Therefore, there is considerable interest in identifying antigens that can induce efficient CD8^+^ T cell responses to improve vaccine efficacy and serve as therapeutic targets.

Cytotoxic CD8^+^ T cells are activated when they recognize a foreign peptide presented in the context of major histocompatibility complex class I (MHC-I, human leukocyte antigen [HLA] in humans) molecules. Algorithms used to predict T cell epitopes are designed and trained on datasets often biased to particular ethnicities, such as Caucasian (28). Of the 638 HBV-derived HLA class I peptides described in Immune Epitope Database & Tools (IEDB) (29), 448 (70.2%) are HLA-A restricted and the majority of these are restricted to HLA-A*02:01 (November 2024). Although HLA-A*02:01 is a common allele in the global population, it is not necessarily frequent in WHO regions where HBV prevalence is highest. Indeed, T cell epitopes frequently found in Caucasian patients were rarely detected in Chinese patients and dominant HBV epitopes may not always be presented by HLA-A molecules (30, 31). Thus, there is a need to identify HBV peptides presented on other MHC-I molecules, particularly HLA-B and HLA-C, which are prevalent in regions with high HBV infection rates (32).

Peptides are selected for presentation by the MHC- I antigen processing and presentation pathway. In this pathway, the peptide loading complex (PLC) consisting of the transporter associated with antigen processing (TAP)1 and 2, calreticulin, ERp57 and tapasin, stabilizes peptide-receptive MHC class I and provides it with a peptide (33–36). The peptide-editing activity of tapasin was shown to affect the presentation of viral peptides in mice (37–39). The tapasin homologue TAPBPR can perform MHC class I peptide exchange outside the context of the PLC to optimize the peptide repertoire ultimately presented at the cell surface for immunosurveillance (40–43). This peptide editor can perform direct peptide exchange or recruit UDP-glucose: glycoprotein glucosyltransferase 1 (UGGT1), which reglucosylates peptide-receptive MHC class I and recycles it back to the PLC for tapasin-dependent peptide acquisition (44, 45). Structural studies have revealed that TAPBPR mediates peptide exchange by widening and stabilizing the MHC-I peptide binding groove (46, 47). TAPBPR has an editing loop region, comprising residues K22-D35, that is in proximity to the MHC-I F pocket. This editing loop has been proposed to dissociate peptides by inserting into the MHC-I F pocket and/or promote peptide loading by hovering above the cleft and drawing a peptide into the MHC class I F pocket (48–52). While recombinant TAPBPR has been shown to promote exogenously added viral peptide loading onto plasma membrane-expressed MHC-I in *in vitro* assays (53), a role for endogenously expressed TAPBPR in the presentation of endogenous viral peptides has not been demonstrated to date.

Our aim was to characterize HBV-derived peptides that are presented on selected HLA-B and HLA-C molecules that are prevalent in regions with high rates of HBV infection and HBV-related HCC mortality (32). We were particularly interested in HLA-B*08:01, -B*35:01, and -C*06:02. HLA-B*08:01 and -B*35:01 represent two of the most common HLA-B alleles world-wide and present in high frequency in the WHO Western Pacific region (32). However, only 10 and 9 HBV-derived peptides have been described for these allotypes, respectively (29). HLA-C*06:02 is the most common HLA-C allele in the WHO African region (32) and no HLA-C*06:02-restricted HBV-derived peptides have been described. Here, using single allele cell lines, we identify novel HBV-derived peptides presented on these HLA class I molecules, as well as on endogenously expressed HLA-A*68:02, -B*15:03, and -C*12:03. Additionally, we explore the role of TAPBPR in the selection of HBV-derived peptides for presentation on HLA class I molecules.

## 2 Materials & methods

### 2.1 Plasmids

Single guide RNAs (sgRNAs) specific for HLA-A (ACAGCGACGCCGCGAGCCAG), HLA-B (GGTTCTATCTCCTGCTGGTC), HLA-C (CGGACTGGTCATACCCGCGG) (54) or TAPBPR (GCGAAGGACGGTGCGCACCG) (42) were encoded by pSpCas9(BB)-2A-Puro (PX459, Addgene plasmid 48139). HLA class I genes were cloned into the lentiviral vector pHRSINcPPT-SGW as previously described (55). FLAG-tagged HBsAg, Pol, HBx (amplified from Addgene plasmids 103011, 65520, 65463) or HBcAg (purchased from Eurofins Genomics) were cloned into lentiviral vector pHRSINcPPT-SGW.

### 2.2 Cell culture

HeLa (ATCC), HeLaM [a variant of the HeLa cell line that is more responsive to interferons (56)] and human embryonic kidney 293T (HEK-293T, kind gift from Paul Lehner, University of Cambridge) cells were cultured in Dulbecco’s Modified Eagle’s Medium (DMEM, Gibco) supplemented with 5-10% Fetal Bovine Serum, (FBS, Gibco), 100U/mL penicillin & 100μg/mL streptomycin (Pen/Strep, Gibco) at 37°C with 5% CO_2_. Where indicated, cells were treated with 200U/mL IFN-γ (Peprotech) for 48-72h.

### 2.3 Depletion of HLA class I or TAPBPR using CRISPR/Cas9 transfection

HeLa HLA-A, -B, -C knock-out (ABC^KO^) or TAPBPR^KO^ cells were generated by transfection as described previously (42, 57). Briefly, HeLa cells were transfected with PX459 containing HLA class I or TAPBPR sgRNAs using Lipofectamine 2000 (Invitrogen). 24h after transfection, the medium was replaced with culture medium supplemented with 4 µg/ml puromycin (Invivogen). After 48h, the media was replaced with culture medium without puromycin. Single cell KO clones were obtained using limiting dilution.

### 2.4 Lentiviral transduction of HLA class I and HBV proteins

Lentivirus was produced in HEK-293T cells by transfecting HEK-293T cells with 0.8μg pHRSINcPPT-SGW encoding the protein of interest, along with 0.5μg packaging vector pCMVΔR8.91 and 0.5μg envelope vector pMD2.G using FuGENE (Promega). Supernatant containing lentivirus was harvested 48h and 72h post-transfection, and centrifuged, filtered, supplemented with 8μg/mL polybrene (Sigma-Aldrich), and added to the target cells for transduction. HeLa HLA-ABC^KO^ cells were transduced with one of the indicated HLA class I constructs, and subsequently with one of the HBV protein-encoding plasmids. Similarly, HeLa expressing endogenous TAPBPR (TAPBPR^WT^) or TAPBPR^KO^ cells were transduced with individual HBV constructs.

### 2.5 Immunoprecipitation of TAPBPR

IFN-γ-stimulated cells were lysed with 1% Triton X-100 (VWR) in Tris-buffered saline (TBS) consisting of 20mM Tris-HCl, 150mM NaCl and 2.5mM CaCl_2_ supplemented with 10mM N-ethylmaleimide (NEM, Sigma-Aldrich), 1mM phenylmethylsulfonyl fluoride (PMSF, Sigma-Aldrich), and a Complete mini EDTA-free protease inhibitor cocktail tablet (Roche). Nuclei and cell debris were pelleted by centrifugation and supernatants were incubated with 5 μg PeTe4 conjugated to protein A Sepharose beads (Generon) for 90 min at 4 °C. Beads were washed three times in 0.1% Triton X-100 in TBS and resuspended in 125 mM Tris-HCl pH6.8, 4% SDS (Melford), 20% glycerol (Fisher Chemical), 0.04% bromophenol blue (Sigma-Aldrich), supplemented with 100 mM β-mercaptoethanol (Sigma-Aldrich).

### 2.6 Validation of HLA class I, HBV protein or TAPBPR levels by immunoblotting

To prepare whole cell lysates, samples were lysed in 1% Triton X-100 in 20 mM Tris-HCl, 150 mM NaCl, 2.5 mM CaCl_2_, supplemented with 10 mM NEM, 1 mM PMSF and a Complete mini EDTA free protease inhibitor cocktail tablet for 30 minutes at 4 °C. Immunoprecipitation samples or whole cell lysates were separated by SDS-PAGE followed by transfer to an immobilon transfer membrane (Merck Millipore). Membranes were blocked using 5% (w/v) dried milk in PBS, stained with 0.5 μg/ml mouse anti-FLAG (Sigma Aldrich), 0.4 μg/ml mouse anti-TAPPR (abcam), 1:1000 HCA2 (in house), 1:1000 HC10 (in house), or 0.5 μg/ml rabbit anti-calnexin (Enzo) followed by 1:5000 goat anti mouse-HRP (DAKO) in PBS supplemented with 5% dried milk and 0.05% Tween-20 (Sigma). Proteins were detected by enhanced chemiluminescence using Western Lightning (Perkin Elmer) and Super RX film (Fujifilm). Films were scanned on a CanoScan8800F using MX Navigator Software (Canon).

### 2.7 Validation of HLA class I or TAPBPR expression by flow cytometry

Surface HLA class I was stained using 3-33 μg/ml mouse DT9 followed by 2μg/ml goat anti-mouse Alexa Fluor 647 (Invitrogen) or using 2.5 μg/ml W6/32-PB (Biolegend), 1:50 BB7.2-AF647 (ThermoFisher Scientific), 1:50 Bw6-APC (Miltenyi Biotec) in PBS supplemented with either 1% bovine serum albumin (BSA, Cytiva) or 2% FBS. For intracellular staining, cells were fixed for 15 minutes at room temperature using 4% paraformaldehyde (VWR Chemicals) in PBS. Cells were incubated in blocking buffer containing in PBS-EDTA supplemented with 40% human serum (Sigma-Aldrich), 4% fetal calf serum (FCS, Gibco) and 0.2% saponin (Sigma). Cells were stained with 0.5 μg/ml PeTe4, 1:1000 W6/32 (in house), or 1:1000 IgG2a isotype control (Dako) followed by 2μg/ml goat anti-mouse Alexa Fluor 647 (Invitrogen) in PBS-EDTA with 4% FCS and 0.2% saponin. Fluorescence levels were detected using a Cytek DxP8 flow cytometer (Cytek) or CytoFLEX S or LX flow cytometer (Beckmann Coulter) and analyzed using FlowJo software.

### 2.8 HLA class I immunopeptidomics

Cells transduced with a single HLA allotype or wild-type and TAPBPR^KO^ cells expressing HBsAg/Pol or HBxAg/HBcAg were pooled to obtain 10^8^ cells/sample (one biological replicate). Cells were lysed in a 1.2% (w/v) CHAPS-based lysis buffer and HLA class I molecules were isolated from samples by standard immunoaffinity purification (58) using the pan-HLA class I-specific monoclonal antibody W6/32 in Econo Column^®^ Chromatography Columns (0.5cm x 5cm or 5cm x 5cm BioRad), followed by acidic elution with 0.2% (v/v) trifluoro acetic acid (TFA), size-exclusion filtration with an Amicon Ultra 0.5 centrifugal filter unit (Merck Millipore) and a subsequent desalting step using a ZipTip C18 pipette tip (Merck-Milipore). The eluted peptides were analyzed in three technical replicates for qualitative analysis and in five technical replicates for relative quantification as described previously (59, 60). Peptides were separated by nanoflow high-performance liquid chromatography. Eluted peptides were analyzed in an online-coupled Orbitrap Fusion Lumos mass spectrometer (Thermo Fisher). Spectra were annotated to corresponding peptide sequences by database search across the human proteome as included in the Swiss-Prot database. The Proteome Discoverer (v1.3, Thermo Fisher) was used to integrate the search results of the SequestHT search engine (61) against the human proteome (Swiss-Prot database, 20279 reviewed protein sequences, 27 September 2013), the complete HBV proteome (GenBank KY003230), and HBV proteomes from genotype A-H (GenBank EU054331, AB219428, GQ924620, FJ904433, AY738145, AY090458, AB064313, FJ356716). Precursor mass tolerance was set to 5 ppm. Fragment mass tolerance was set to 0.02 Da. Peptide length was restricted to 8–12 amino acids and oxidized methionine was allowed as dynamic modification. The FDR estimated by the Percolator algorithm 2.04 (62) was limited to 5%. A second layer of quality criteria was applied using HLA class I annotation with SYFPEITHI 1.0 (63) and NetMHCpan 4.0 (64). Only predicted binders were included in the analysis. Relative quantification of HLA ligands was performed based on the area of the corresponding precursor extracted ion chromatograms using the Proteome Discoverer (v1.4, Thermo Fisher). For Label-free quantitation analysis (LFQ), the total injected peptide amount of all samples was normalized prior to LC-MS/MS analysis.

### 2.9 Bioinformatic analyses

Peptide length was restricted to 8–12 amino acids and peptide lists of predicted binders were processed using original Python 3.7 code using NumPy (65), pandas (66), matplotlib (67), seaborn (68), and logomaker (69) packages. Fold-changes for volcano plots were calculated using an in-house R script (v3.2) to show pairwise comparisons of the ratios of the mean areas for each individual ligand in the five LFQ-MS runs. Significant modulation was defined by an adjusted p-value of <0.01 and ≥log2 2-fold change, as calculated by two-tailed t-tests implementing Benjamini-Hochberg correction.

### 2.10 HBV peptide binding to HLA class I

HeLa HLA-ABC^KO^ or single HLA class I allotype-expressing cells were incubated for 1h with 1 or 10 μM lysine^TAMRA^ (K*)-labelled peptide (Peptide Synthetics) in Opti-MEM (Gibco) at 37°C. Cells were harvested using 0.05% EDTA-trypsin (Gibco) and stained with 1 μg/ml mouse-anti-TAMRA (Abcam) followed by 2 μg/ml goat anti-mouse-AF647 (Invitrogen). TAMRA-positive cells were detected using a CytoFLEX LX flow cytometer (Beckmann Coulter) and analyzed using FlowJo software.

### 2.11 TAPBPR peptide exchange assay

Wild-type HeLaM cells, or cells transduced to overexpress TAPBPR^WT^ or TAPBPR^Øloop^ (50) were IFN-γ-treated. The cells were incubated for 15min at 37°C with 10 or 100 nM lysine^TAMRA^-labelled peptide in Opti-MEM. Cells were harvested using 0.05% EDTA-trypsin and stained with 0.3 μg/ml PeTe6-AF680 (in house) in PBS with 2% FBS. Samples were analyzed using a CytoFLEX LX flow cytometer and FlowJo software.

### 2.12 Statistical analyses

Binding of TAMRA-labelled peptide to HLA class I was compared using a paired t-test. Fold-change of peptide abundance in the presence or absence of TAPBPR was analyzed using a two-tailed t-tests implementing Benjamini-Hochberg correction. HLA class I expression levels after transduction were analyzed using a one-Way ANOVA (mixed-effects analysis and Dunnett’s multiple comparisons test, with single pooled variance).

## 3 Results

### 3.1 Identification of novel HBV-derived peptides presented on HLA-A*02:01, -B*08:01, -B*35:01 and -C*06:02

To discover novel HBV-derived peptides presented on specific HLA class I, HeLa cells lacking endogenous HLA-A, -B, and -C were generated (**Supplementary Figure 1A**), then transduced to express either HLA-A*02:01, -B*08:01, -B*35:01, or -C*06:02 (**Supplementary Figure 1B**). Subsequently, the individual HBV open reading frames (ORFs) HBsAg, Pol, HBcAg, or HBxAg were expressed in the cells (**Supplementary Figure 1C**).

**Figure 1:**
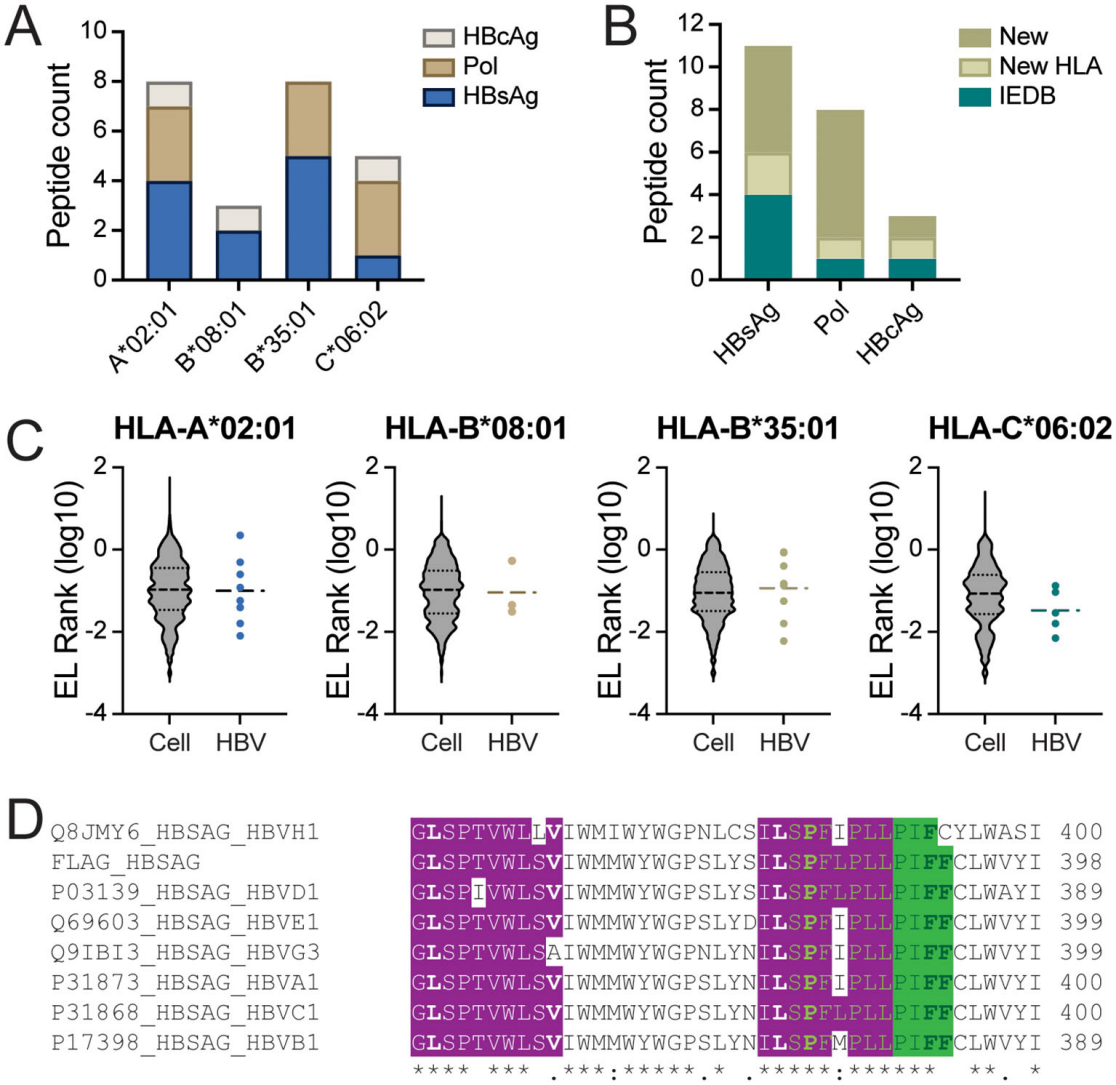
Identification of novel HBV-derived peptides presented on HLA-A*02:01, -B*08:01, -B*35:01 and -C*06:02. Peptides were eluted from W6/32-reactive HLA complexes isolated from HeLa HLA-ABC^KO^ cells transduced to express one of the indicated HLA allotypes in combination with either HBsAg or Pol, or HBxAg or HBcAg. The peptide repertoire was established using mass spectrometry. **(A)** Number of HBV-derived peptides per HLA allotype. **(B)** Number of peptides previously described in IEDB (29) and novel peptides per HBV antigen. ‘New HLA’ indicates peptide sequences previously described in the IEDB, however, not on the HLA class I molecule it was detected on. **(C)** The predicted affinity (EL Rank) of each peptide for their respective HLA class I allotype was predicted using panNetMHC4.0 (64). Median and for cellular peptides also quartiles are shown. **(D)** Fragment of HBV genotype A-H HBsAg sequences obtained from Uniprot (accession numbers indicated) aligned with the FLAG-tagged sequence used to transduce HeLa cells using Clustal Omega (70) illustrating conservation of anchor residues. Peptides detected by immunopeptidomics are highlighted. Bold = expected anchor residue. (**Supplementary Figures 1–5** and http://mhcmotifatlas.org).

Immunopeptidomic analysis of these cells was then used to identify peptides presented on the transduced HLA class I. Our immunopeptidome datasets were validated by performing SeqLogo analysis of the total immunopeptidome derived from our cell lines, which confirmed the expected anchor residues for each HLA class I allotype (**Supplementary Figure 2**). Next, we explored the immunopeptidomes for HBV-derived presented peptides, with the advantage that single HLA class I allele-expressing cell lines enable peptide discovery without the need for algorithm-based assignment of peptides to one of several endogenously expressed alleles. Using this approach, we identified 22 HBV-derived peptide sequences presented on the four HLA class I molecules: 8 peptides presented on HLA-A*02:01, 3 on HLA-B*08:01, 8 on HLA-B*35:01 and 5 on HLA-C*06:02 (**Table 1**; **Figure 1A**). Eleven of the identified HBV peptides were from HBsAg, 8 from Pol and 3 from HBcAg (**Table 1**; **Figure 1B**). Of note, we did not identify any peptides derived from HBxAg.

**Table 1:** HBV-derived peptides presented on HLA-A*02:01, -B*08:01, -B*35:01 or -C*06:02.

Protein	Length	Sequence*	IEDB**	Allele ***	EL Rank ***	Q-value	Xcorr
HBcAg	10	FLPSDFFPSV	YES	A*02:01	0.12	0	2.991
HBsAg	10	GLSPTVWLSV	YES	A*02:01	0.251	0.001	3.130
HBsAg	9	ILSPFLPLL	YES	A*02:01	0.016	0.007	2.043
HBsAg	9	GmLPVCPLI	YES	A*02:01	0.49	0.001	2.682
Pol	11	SLDVSAAFYHL	NO	A*02:01	2.223	0	4.392
Pol	9	SLYADSPSV	YES	A*02:01	0.008	0.001	2.321
HBsAg	9	FLLTRILTI	YES	A*02:01	0.039	0.004	2.368
			B*08:01 new	B*08:01	0.045	0.003	2.771
Pol	9	HLYSHPIIL	YES	A*02:01	0.057	0.004	2.411
			C*06:02 new	C*06:02	0.133	0	2.563
HBcAg	9	NMGLKIRQL	NO	B*08:01	0.031	0.002	2.627
HBsAg	9	SARFSWLSL	NO	B*08:01	0.536	0.016	2.072
HBsAg	9	IPIPSSWAF	YES	B*35:01	0.006	0.004	2.511
HBsAg	10	SPFLPLLPIF	NO	B*35:01	0.399	0.006	1.885
Pol	10	TPSRVTGGVF	NO	B*35:01	0.848	0.003	1.514
Pol	9	QAFTFSPTY	NO	B*35:01	0.016	0	3.006
HBsAg	9	QPTPLSPPL	NO	B*35:01	0.136	0.031	2.140
HBsAg	11	SPFLPLLPIFF	NO	B*35:01	0.875	0	3.122
HBsAg	9	TASPLSSIF	B*35:01 new	B*35:01	0.056	0.004	2.369
Pol	10	YPEHLVNHYF	NO	B*35:01	0.15	0	2.782
HBsAg	9	ARFSWLSLL	NO	C*06:02	0.029	0	2.211
Pol	9	SRNLYVSLL	NO	C*06:02	0.016	0.002	2.310
HBcAg	9	YRPPNAPIL	C*06:02 new	C*06:02	0.007	0.002	2.185
Pol	9	VSIPWTHKV	NO	C*06:02	0.093	0.029	1.178

*Anchor residues described at http://mhcmotifatlas.org are underlined. **IEDB (29) as of November 2024. ***Based on NetMHCpan 4.0 (64).

To determine whether the identified peptides had been described before, we screened them against the IEDB (29) and defined 3 categories: novel, known, and known but novel for the HLA class I allotype that we identified it on. Eight of the peptides identified were previously characterized HBV-derived HLA class I peptides: 7 of which were known to be presented on HLA-A*02:01 and one was already characterized as an HLA-B*35:01 binder (**Table 1**; **Figure 1B**). This confirmation of known peptides in our datasets was reassuring, particularly for HLA-A*02:01, which we included here as a positive control. Additionally, 4 of the HBV-derived peptides we identified were in the IEDB but assigned to different HLA molecules. Strikingly, 11 of the HBV-derived peptides we identified appeared to be novel and not currently in the IEDB (**Table 1**; **Figure 1B**). When we determined the predicted affinity of the identified HBV-derived peptides for their respective HLA molecules, it was similar to that of the cellular-derived peptide repertoire (**Figure 1C**). Taken together, by performing immunopeptidomic analysis on cells expressing HBV-derived proteins, we have identified novel HBV peptides presented on HLA class I.

### 3.2 HBV-derived peptides have conserved anchor residues

Next, we mapped the identified HBV-derived peptides onto the HBV proteins expressed in the cell and additionally included the protein sequence from HBV genotypes A-H. First, this revealed that the identified peptides were derived from across the entire coding regions (**Supplementary Figures 3**–**5**). Furthermore, the anchor residues of the detected HBV-derived peptides were typically highly conserved across HBV genotypes (**Supplementary Figures 3**–**5**). For example, despite the variation in the HLA-A*02:01-restricted peptide GLSPTVWLSV between HBV genotypes, the C-terminal residue was conserved in all but one genotype. Additionally, the anchor residue at position 2 was observed in all eight genotypes (**Figure 1D**; **Supplementary Figure S2**). Furthermore, in protein regions that show high conservation across HBV genotypes, we frequently detected overlapping peptides presented on the same or different allotypes, e.g. the HLA-A*02:01-restricted ILSPFLPLL and the HLA-B*35:01-restricted SPFLPLLPIF(F) (**Figure 1D**; **Supplementary Figure S3**-**5**). Virtually all HBV-derived peptides have one or both anchor residues conserved in the majority, if not all, HBV genotypes (**Supplementary Figures 3**–**5**), indicating that mutations in these regions may impact viral fitness.

### 3.3 Validation of HLA class I-binding of newly identified HBV-derived peptides

**Figure 2:**
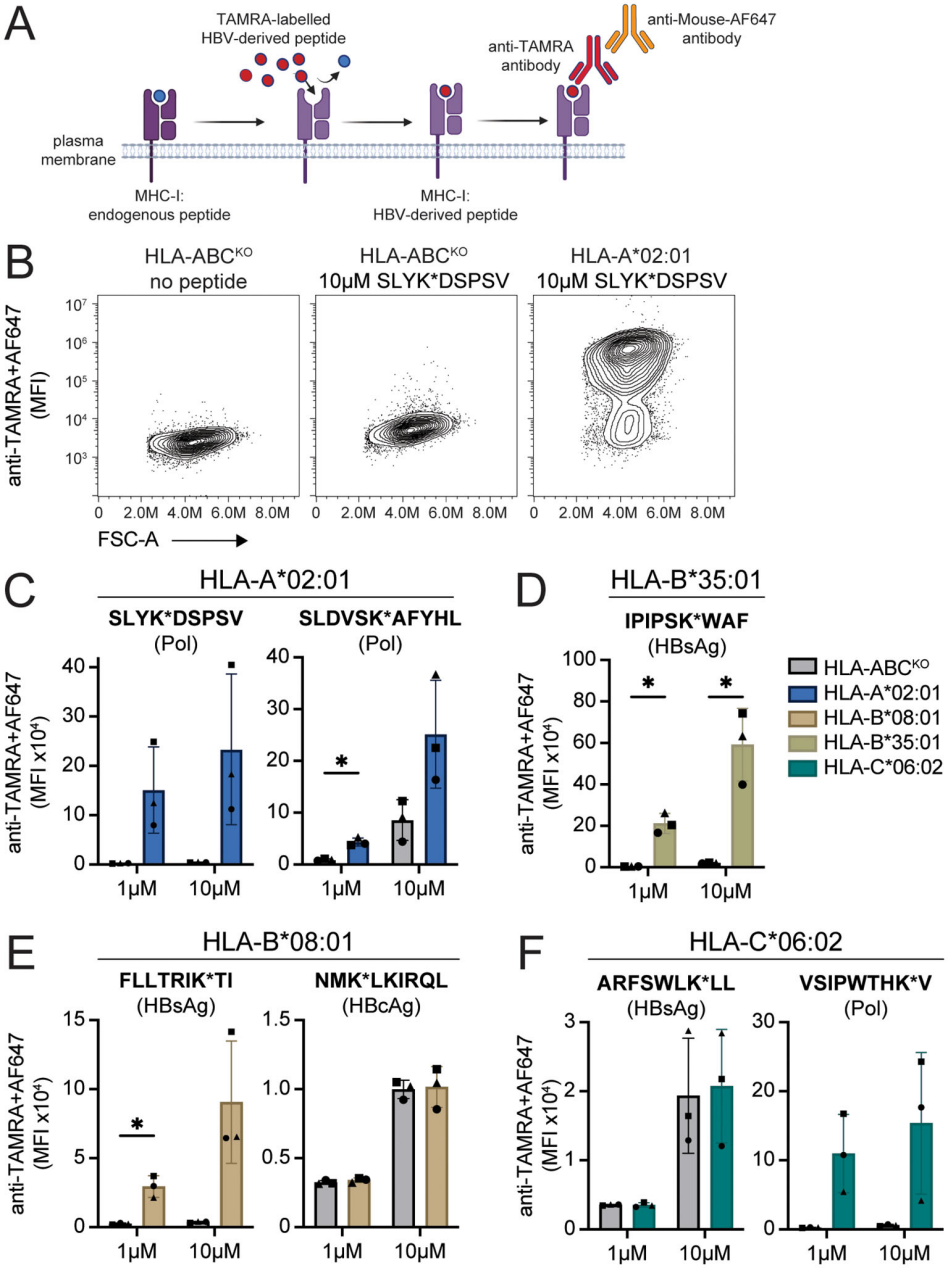
Validation of HLA class I-binding of HBV-derived peptides. **(A)** Schematic overview of peptide pulse assay. Created in BioRender, licensed under CC BY 4.0. **(B)** Representative flow cytometry plots showing the anti-TAMRA antibody stain as detected with anti-Mouse-AF647 on HeLa HLA-ABC^KO^ or HLA-A*02:01 transduced cells without peptide or with 10μM SLYK*DSPSV. **(C-F)** HeLa HLA-ABC^KO^ cells untransduced (negative control) or transduced with HLA-A*02:01 **(C)**, -B35*01 **(D)**, -B*08:01 **(E)**, or C*06:02 **(F)** were pulsed with 1 or 10μM of the indicated TAMRA-labelled peptide. Peptide binding at the cell surface was measured using an anti-TAMRA stain. Mean and standard deviation of three biological repeats is shown. Paired t-test. Absence of asterisk indicates p>0.05, i.e. not statistically significant. *p<0.05.

To validate our discovery approach, 7 of the identified HBV-derived HLA class I peptides were selected to confirm their binding to their respective HLA class I allotype via peptide pulsing (**Supplementary Table 1**). To permit peptide binding detection using flow cytometry, a fluorescent TAMRA label was incorporated into the peptides at a position deemed not to interfere with anchor binding to the MHC-I molecule. A surface stain with an anti-TAMRA antibody was used as a readout for peptide binding (**Figures 2A, B**), thereby eliminating any background signal arising from endocytosis of TAMRA-labelled peptides.

As positive controls, we tested the binding of peptides SLYK*DSPSV (HLA-A*02:01) and IPIPSK*WAF (HLA-B*35:01), previously described in IEDB (29). We confirmed that SLYK*DSPSV bound to HeLa cells expressing HLA-A*02:01 but not its HLA-ABC^KO^ counterpart (**Figure 2C**). Similarly, IPIPSK*WAF bound to HLA-B*35:01-expressing HeLa cells but not to HLA-ABC^KO^ cells (**Figure 2D**). For the novel HBV-derived peptides, we confirmed the specific binding of SLDVSK*AFYHL to HLA-A*02:01 (**Figure 2C**), FLLTRIK*TI to HLA-B*08:01 (**Figure 2E**), and VSIPWTHK*V to HLA-C*06:02 (**Figure 2F**), demonstrating the validity of our discovery approach. However, the HBV-derived peptides NMK*LKIRQL (identified in the immunopeptidome of HLA-B*08:01 expressing cells) and ARFSWLK*LL (detected in the immunopeptidome of HLA-C*06:02 expressing cells) were detected at lower levels and these were equal between HLA-ABC^KO^ and HLA class I-transduced cells (**Figures 2D, F**). This suggests these peptides may bind something other than HLA-A, -B or -C at the cell surface.

### 3.4 Discovery of novel HBV-derived peptides presented on HLA-A*68:02, -B*15:03 and -C*12:03

**Figure 3:**
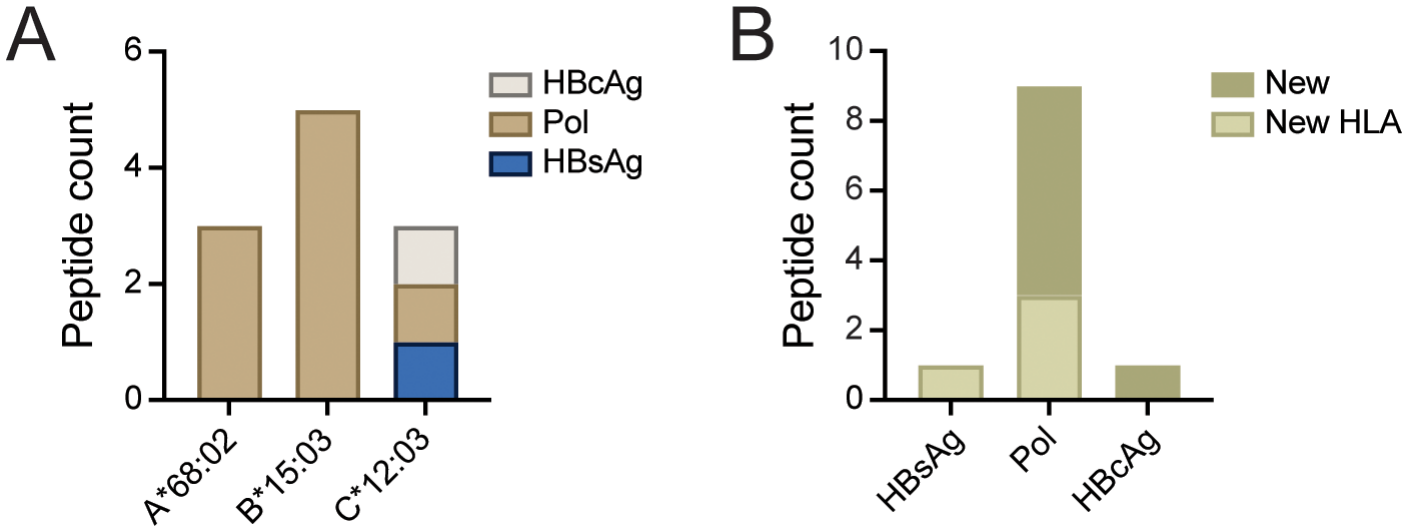
HBV-derived peptides are presented on HeLa cells. Wild-type HeLa cells transduced to express either HBsAg, Pol, HBxAg or HBcAg were treated with IFN-γ. Peptides were eluted from W6/32-reactive HLA class I complexes and the peptide repertoire was established using mass spectrometry. **(A)** Peptides were assigned to one of the indicated HLA class I allotypes using panNetMHC4.0 (64). Number of HBsAg, Pol or HBcAg-derived peptides per HLA allotype. **(B)** Number of peptides previously described in IEDB (29) and novel peptides per HBV antigen. ‘New HLA’ indicates peptide sequences previously described in the IEDB, however, not on the HLA class I molecule it was assigned to.

To expand our HBV-derived peptide discovery to additional MHC-I molecules, we expressed the HBV-derived proteins HBsAg, Pol, HBcAg or HBxAg in wild-type HeLa cells, which endogenously express HLA-A*68:02, -B*15:03 and -C*12:03 (**Supplementary Figure 6**). Following immunopeptidomic analysis of peptides isolated from MHC-I molecules from these cells, the peptides were assigned to any of the three allotypes using panNetMHC4.0. The overall peptide repertoire showed the expected anchor residues for each allotype (**Supplementary Figure 7**), confirming the successful isolation and assignment of peptides to specific MHC-I molecules. In the immunopeptidomes from these cells, we identified 11 HBV-derived peptide sequences presented on the three HLA molecules: 3 peptides presented in HLA-A*68:02, 5 on HLA-B*15:03 and 3 on HLA-C*12:03 (**Table 2**; **Figure 3A**). Nine of the identified HBV peptides were derived from Pol, 1 was from HBsAg and 1 was from HBcAg (**Table 2**; **Figure 3B**). We did not identify any peptides derived from HBxAg. All the identified HBV-derived peptides were either not described before or described as binding to a different allotype than the one on which we detected them (**Figure 3B**; **Table 2**).

**Table 2:** HBV-derived peptides eluted from endogenously expressed HLA-A*68:02, -B*15:03 or -C*12:03.

Protein	Length	Sequence *	IEDB**	TAPBPR	Allele ***	EL Rank ***	Q-value	Xcorr
Pol	9	DVSAAFYHL	NO	WT & KO	A*68:02	0.046	0	3.136
Pol	9	FTAVTNFLL	NO	WT & KO	A*68:02	0.17	0	3.478
Pol	9	FTFSPTYKA	A*68:02 new	KO	A*68:02	0.014	0	2.449
Pol	9	FTSAICSVV	A*68:02 new	WT & KO	A*68:02	0.299	0.006	2.327
Pol	9	AAYPAVSTF	NO	WT & KO	B*15:03	0.005	0	2.879
Pol	9	IQSKQAFTF	NO	WT & KO	B*15:03	0.019	0	3.436
Pol	10	KQAFTFSPTY	NO	WT & KO	B*15:03	0.035	0	4.169
HBcAg	9	LKIRQLLWF	NO	KO	B*15:03	1.18	0	2.083
Pol	9	RAFPHCLAF	B*15:03 new	WT & KO	B*15:03	0.029	0	2.641
Pol	9	RKIPmGVGL	NO	WT & KO	B*15:03	0.212	0.001	2.155
Pol	11	YKRETTHSASF	NO	KO	B*15:03	0.232	0.038	2.183
HBcAg	9	FGRETVLEY	NO	WT & KO	C*12:03	0.069	0	2.655
Pol	9	HLYSHPIIL	C*12:03 new	WT & KO	C*12:03	0.143	0	2.635
HBsAg	9	SARFSWLSL	NO	KO	C*12:03	0.441	0.013	1.980
HBsAg	9	TASPLSSIF	C*12:03 new	WT & KO	C*12:03	0.059	0.001	2.727

*Anchor residues described at http://mhcmotifatlas.org are underlined. **IEDB (29) as of November 2024. ***Based on NetMHCpan 4.0 (64).

### 3.5 TAPBPR shapes the presentation of some HBV-derived peptides

As the effect of the peptide editor TAPBPR on the presentation of virus-derived peptides within a cellular environment has not been investigated to date, we assessed its role in the presentation of HBV-derived peptides. To this end, HeLa cells were chosen for this work as these cells express HLA-A*68:02 which is the strongest known binder of TAPBPR (55). TAPBPR was knocked out in HeLa cells (**Supplementary Figures 6A, B**), which did not substantially affect HLA class I levels (**Supplementary Figure 6C**). HBsAg, Pol, HBcAg, or HBxAg were then introduced into these cells (**Supplementary Figure 6D**).

**Figure 4:**
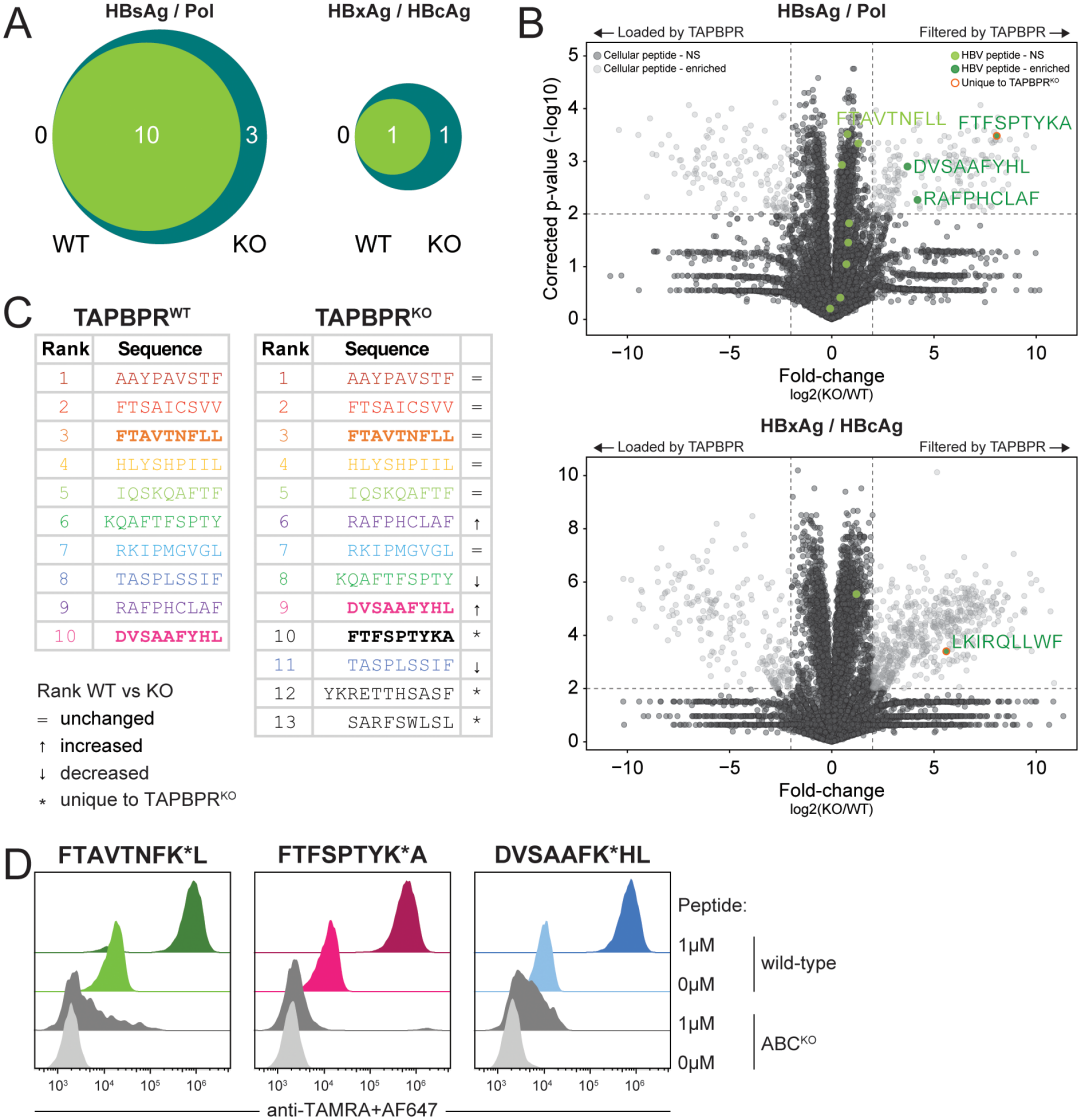
TAPBPR-mediated restriction of the HBV-derived peptide repertoire on HeLa cells. Cells were treated with 200U/mL IFN-γ (Peprotech) for 48-72h. Peptides were eluted from W6/32-reactive HLA complexes isolated from wild-type (WT) or TAPBPR^KO^ HeLa cells transduced with HBsAg, Pol, HBxAg or HBcAg. The peptide repertoire was established using mass spectrometry. **(A)** Size and overlap of the HBV-derived peptide repertoire eluted from TAPBPR^WT^ or TAPBPR^KO^ HeLa cells expressing either HBsAg/Pol or HBxAg/HBcAg. **(B)** Semi-quantitative analysis of peptide abundance. In the HbxAg/HBcAg plot (bottom), a single non-significant cellular peptide was cut off the x-axis. **(C)** HBV-derived peptides were ranked according to their abundance in either TAPBPR^WT^ or TAPBPR^KO^ HeLa cells. **(D)** IFN-γ-treated wild-type or HeLaM HLA-ABC^KO^ (also TAPBPR^KO^) cells were pulsed with 1μM of the indicated TAMRA-labelled peptide. Peptide binding at the cell surface was measured using an anti-TAMRA stain.

Comparison of the immunopeptidome presented on TAPBPR^KO^ HeLa cells to cells with endogenous TAPBPR expression revealed a small expansion in HBV-derived peptide repertoire presented on HLA class I in the absence of TAPBPR (**Figure 4A**; **Table 2**). This is in line with the previous observation that TAPBPR activity in HeLaM cells restricts the cellular peptide repertoire (42, 50, 52). Specifically, four HBV-derived peptides were only detected in TAPBPR knockout HeLa cells: FTFSPTYKA (derived from Pol and predicted to be presented on HLA-A*68:02), LKIRQLLWF (derived from HBcAg, predicted presentation on HLA-B*15:03), YKRETTHSASF (derived from Pol, predicted presentation on HLA-B*15:03) and SARFSWLSL (from HBsAg with predicted presentation on HLA-C*12:03) (**Table 2**). The peptides unique to TAPBPR^KO^ cells were considered to be blocked or removed by TAPBPR, as they were detected only in the absence of the peptide editor.

As our datasets included semi-quantitative data, we were further able to assess the impact of TAPBPR on the relative abundance of HBV-derived peptides (**Figure 4B**). Of the three HBsAg/Pol peptides that were statistically significantly filtered by TAPBPR, only FTFSPTYKA was uniquely detected in TAPBPR^KO^ cells (**Figures 4A, B**; **Table 2**). The other two peptides (DVSAAFYHL and RAFPHCLAF) were presented regardless of TAPBPR; however, in its presence, they were less abundant (**Figure 4B**). The single HBcAg-derived peptide that was filtered by TAPBPR (LKIRQLLWF) was also unique to the TAPBPR^KO^ cells and therefore absent in the presence of TAPBPR (**Figures 4A, B**; **Table 2**). Taken together, TAPBPR can restrict presentation of some HBV-derived peptides on HLA class I allotypes expressed by HeLa cells.

Comparison of the HBV peptide abundance ranking showed that the 5 most abundant peptides did not change rank in the presence or absence of TAPBPR (**Figure 4C**). Furthermore, the peptides filtered by TAPBPR were of relatively low abundance and ranked at the bottom of the table (**Figure 4C**). Interestingly, while two HBV-derived peptides (RAFPHCLAF and DVSAAFYHL) were more abundant in cells lacking TAPBPR, two peptides (KQAFTFSPTY and TASPLSSIF) were relatively more abundant in cells with TAPBPR (**Figure 4C**).

We tested the ability of three TAMRA-labelled derivatives of the identified HBV-pol-derived peptides predicted to bind to HLA-A*68:02 to bind to MHC-I in high-dose peptide pulse experiments (**Supplementary Table 2**; **Figure 4C**). FTAVTNF*L, FTFSPTYK*A, and DVSAAFK*HL all bound to plasma membrane-expressed MHC-I on HeLa cells in peptide-pulse experiments (**Figure 4D**), further validating our HLA class I peptide discovery.

### 3.6 TAPBPR can load HBV-derived peptides on HLA class I

**Figure 5:**
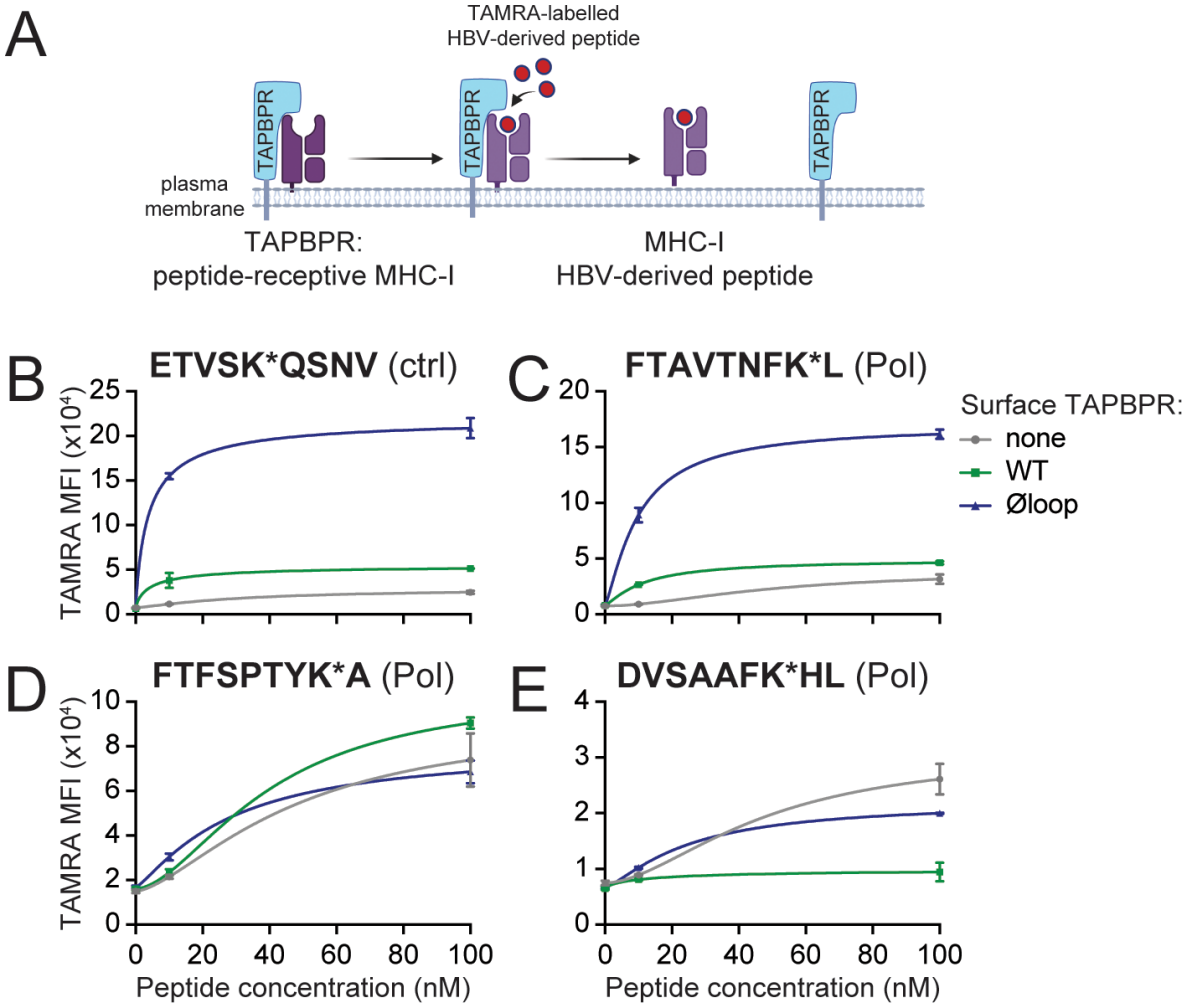
TAPBPR selects HBV-derived viral peptides for presentation on HLA class (I) **(A)** Schematic overview of TAPBPR peptide exchange assay. Created in BioRender, licensed under CC BY 4.0. **(B–E)** Wild-type HeLaM cells (no surface TAPBPR) or HeLaM transduced with TAPBPR^WT^ or TAPBPR^Øloop^ (mutated K22-D35 editing loop (50)) were treated with IFN-γ. Cells were incubated for 15min with either 10nM or 100nM of the indicated peptide (**Supplementary Table S2**). K* indicates lysine^TAMRA^. For TAPBPR^WT^ or TAPBPR^Øloop^, equal TAPBPR levels were gated (**Supplementary Figure 8**) before analyzing TAMRA levels. Data is shown as mean+SD of 3 technical repeats with non-linear regression (curve fit). Results are representative of 3 biological repeats.

Overexpression of TAPBPR in HeLa cells causes TAPBPR to traffic to the cell surface, where it can mediate peptide exchange on surface HLA class I (53) (**Figure 5A**), particularly on HLA-A*68:02 due to its strong interaction with TAPBPR (55). We utilized this phenomenon to assess TAPBPR’s ability to load the three selected HLA-A*68:02-restricted HBV-Pol-derived TAMRA-labelled peptides (**Supplementary Table 2**). HeLa cells without TAPBPR at the cell surface were used to establish background levels of peptide binding at low concentrations (**Figures 5B–E**, grey line) and were compared to cells expressing surface TAPBPR^WT^ (**Figures 5B–E**, green line). Although the abundance of FTAVTNFLL was not impacted by TAPBPR expression in our immunopeptidomic studies (**Figure 4C**), TAPBPR was capable of promoting the binding of a TAMRA-labelled derivative of this peptide onto surface MHC-I molecules to a similar extent as the positive control peptide ETVSK*QSNV (a HLA-A*68:02-restricted neoantigen) (**Figures 5B, C**). Surface TAPBPR^WT^ was less efficient at promoting the loading of FTFSPTYK*A, with no enhancement observed above background at 10 nM peptide and only a slight enhancement at 100 nM peptide (**Figure 5D**). The unlabeled variant of this peptide was identified only in cells lacking TAPBPR expression in the immunopeptidomic analysis (**Figure 4**; **Table 2**). Strikingly, for DVSAAFK*HL, surface TAPBPR^WT^ protein did not promote peptide binding (**Figure 5E**). In fact, the TAPBPR^WT^ protein appeared to inhibit the binding of DVSAAFK*HL to MHC-I compared with cells treated with peptide alone (**Figure 5E**). This is in keeping with the immunopeptidomic analysis, which demonstrated that TAPBPR expression restricted the abundance of DVSAAFYHL (**Figure 4**).

### 3.7 The TAPBPR editing loop can contribute to the selection of HBV-derived peptides

TAPBPR has a peptide editing loop in close proximity to the MHC-I F pocket, which can contribute to peptide selection (47, 49–51). As TAPBPR^WT^ blocked DVSAAFK*HL binding to HLA-A*68:02, we next explored whether the editing loop was involved in HBV-derived peptide binding to MHC-I. To this end, we transduced HeLa cells with TAPBPR^Øloop^ in which the loop region was mutated from KDGAHRGALASSED to AAGGSGGGGSGGAA, which was previously shown to render HLA class I highly peptide receptive (50, 52). The transduced cells were gated based on the TAPBPR mean fluorescent intensity (MFI) to ensure comparable surface TAPBPR expression on TAPBPR^WT^ and TAPBPR^Øloop^ transduced cells (**Supplementary Figure 8**). As expected, mutation of the editing loop significantly increased the ability of the control peptide ETVSK*QSNV to bind to MHC-I compared to cells expressing TAPBPR^WT^ (52) (**Figure 5B**) and enhanced binding of the HBV-derived peptide FTAVTNFK*L (**Figure 5C**). For FTFSPTYK*A, the mutation of the editing loop slightly decreased the ability of TAPBPR to load this peptide (**Figure 5D**). Strikingly, upon mutation of the TAPBPR editing loop, the ability of DVSAAFK*HL to bind to MHC-I was restored (**Figure 5E**). This suggests that the TAPBPR editing loop can block this specific HBV-derived peptide from binding to MHC-I and is consistent with the immunopeptidomics observation that TAPBPR expression reduces the abundance of this peptide.

## 4 Discussion

Our aim here was to characterize HBV-derived peptides presented on MHC-I molecules that are prevalent in the WHO African, South and South-East Asian, and Western Pacific regions. Collectively, these regions account for the majority of the global HBV burden yet have been substantially underrepresented in prior epitope discovery efforts. In our study, the extensively characterized HLA-A*02:01 was included as a positive control, while HLA-A*68:02, -B*08:01, -B*15:03, -B*35:01, -C*06:02 and -C*12:03 were used as relevant molecules to reveal novel HBV-derived presented peptides. In antigen processing and presentation-competent cells, we detected 34 HBV-derived peptides presented on various MHC-I molecules, of which 28 were not previously described or not known to bind the HLA class I allotypes we identified. As well as confirming the presence of 7 known HBV-derived peptides on HLA-A*02:01, we also discovered a new peptide presented on this MHC-I molecule. Importantly, 22 of the newly discovered HBV-derived peptides, excluding those that were only presented in the absence of TAPBPR, were presented by HLA-B*08:01, -B*15:03, -B*35:01, -C*06:02 and -C*12:03. This presents a substantial expansion of the 68 HLA-B and 88 HLA-C-restricted HBV-derived peptides described in IEDB.

Most of the HBV-derived peptides we identified were derived from the viral polymerase, followed by HBsAg and HBcAg. This is in line with what has previously been reported (25, 71) and may, in part, be explained by differences in protein length: approximately 850, 400, and 185 amino acids, respectively. We did not detect any HBx-derived peptides, even though they have previously been described in IEDB (29) (72 of 89 [80.9%] HLA-A-restricted, November 2024). With approximately 150 amino acids, HBxAg is of similar size to HBcAg, which limits the chance of producing and detecting HLA class I presented peptides compared to the larger viral polymerase. While HBxAg is a substrate for proteasome degradation, it has also been shown to bind the proteasome alpha subunits and inhibit peptidase activity (72–75). In our HBxAg-transduced cell line, this may hypothetically affect the generation of HBxAg-derived peptides. Furthermore, activation of the immunoproteasome by IFN-γ treatment was shown to be required for the efficient generation of a CD8^+^ T cell epitope derived from HBcAg (expressed from vaccinia virus) (76). As our single HLA cell lines were not IFN-γ-treated, we likely have not detected immunoproteasome-dependent HBV-derived peptides, which may have contributed to the absence of HBxAg-derived peptides in our datasets.

Epitope discovery frequently relies on peptide libraries and on peptide pulsing directly onto MHC molecules (77). While this has proven effective for epitope discovery, it fails to incorporate the effects of the various steps in the antigen processing and presentation pathway, which ultimately determine which peptides make it onto plasma membrane-expressed MHC-I molecules after navigating the intracellular environment. These steps include the degradation of viral proteins into peptides by the proteasome (78), the destruction of peptides by cytosolic peptidases (79), the selection of peptides into the ER lumen via the TAP transporters (80), further peptide trimming in the ER by ERAP1/2 (81) and the selection criteria exerted by the peptide editors tapasin and TAPBPR (35, 42). Thus, by expressing HBV proteins in cells, we have identified peptides that can survive these various degradative and filtration steps, which are likely to be more relevant epitopes in the context of infection than peptides that can simply bind to a particular MHC-I molecule.

Here, we also explored the contribution of the peptide editor TAPBPR to the HBV-immunopeptidome. Previously, TAPBPR has been found to have a restrictive effect on the cellular repertoire (42, 50, 52). Thus, we reasoned that in its absence, the HBV-derived immunopeptidome would be expanded. Indeed, this was the case, with the TAPBPR knockout HeLa cells presenting four new HBV-derived peptides that have not been identified to date. This work represents the first indication that TAPBPR shapes not only the cellular but also the viral peptide repertoire presented on MHC-I molecules. While it may seem counterintuitive for a host to restrict the presentation of pathogen-derived peptides, this may be essential for selecting immunogenic peptides.

Antiviral T cell responses are typically hallmarked by a strong hierarchy, in which most T cells respond to a select few immunodominant peptides (82). CD8^+^ T cells against different HBV epitopes have been shown to exhibit distinct functional properties and the molecular mechanisms underlying impaired T cell responses differ depending on the HBV antigen (23, 83). For example, the frequency and effectiveness of CD8^+^ T cells activated by HBcAg-derived peptides was higher than that of T cells specific for the viral polymerase (83–85). However, host factors and intrinsic abilities of HBV antigens to induce a protective or dominant T cell response remain to be established. Here we showed that while the presentation of the five most abundant HBV-derived peptides did not substantially change in the absence of TAPBPR, the observed changes in the levels of presentation of less abundant peptides may further influence the antiviral CD8^+^ T cell response.

Intriguingly, the plasma membrane-expressed TAPBPR loaded TAMRA-labelled variants of three Pol-derived peptides to different extents onto HLA-A*68:02. While TAPBPR was able to promote the loading of FTAVTNFK*L, it did not particularly enhance the presentation of FTFSDTYK*A onto plasma membrane-expressed HLA-A*68:02. Strikingly, TAPBPR blocked the presentation of DVSAAFK*HL onto HLA-A*68:02. These peptides were all derived from the immunopeptidomics datasets: the presentation of FTAVTNFLL occurred in a TAPBPR-independent manner; FTFSPTYKA was only detected in the absence of TAPBPR; and the abundance of DVSAAFYHL was limited by TAPBPR expression. While TAPBPR is thought to improve the affinity of peptides loaded onto MHC-I (42, 86), the affinity of the three HBV-derived peptides for HLA-A*68:02 does not seem to be the differential factor here, as all three peptides are predicted to be strong binders to HLA-A*68:02 (FTAVTNFLL = 6.50 nM, FTFSPTYKA = 5.35 nM, DVSAAFYHL = 16.5 nM).

We explored whether the TAPBPR editing loop was a contributing factor in determining the degree of peptide loading of these three HBV-derived peptides onto HLA-A*68:02. Our results suggest that the loop region is responsible for limiting peptide loading of these peptides, as mutation of the loop region resulted in higher peptide loading onto MHC-I. Strikingly, the ability of TAPBPR to block DVSAAFK*HL binding seems to be mainly under the control of the editing loop. While we have not assessed the ability of TAPBPR to dissociate the peptides from HLA-A*68:02, the variability in loading of these three peptides by TAPBPR is fascinating and suggests some, as yet, unknown rules to TAPBPR-mediated peptide selection.

While we confirmed the binding of 8 of the 10 selected HBV-derived peptides to their respective HLA class I allotypes as part of this work, we found that two peptides (NMK*LKIRQL and ARFSWLK*LL) bound well to HeLa cells that lack expression of HLA-A, -B, and -C molecules. As these cells still express low levels of W6/32-reactive non-classical HLA-E (also detected using the DT9 antibody [**Supplementary Figure 1C**)], one possibility is that these two peptides may be HLA-E-restricted. Indeed, MHC-E has been shown to activate CD8^+^ T cell responses against HBV (87), which is of particular interest for therapeutic development given the low genetic diversity of HLA-E compared to classical HLA-A, -B, and -C molecules.

A limitation of our study is that the HBV proteins were expressed from transduced HBV ORFs in HeLa cells, with peptide processing potentially differing during a productive infection with associated inflammation in hepatocytes. This approach had the advantage of permitting the identification of peptides in an affinity-independent manner for the single allele work. While for the endogenously expressed HLA-A*68:02, -B*15:03 and -C*12:03 peptide assignment relies on NetMHCpan4.0 prediction algorithms, the HeLa cells have previously been used in most TAPBPR studies as HLA-A*68:02 is the strongest known TAPBPR-binder (55).

The discovery of a substantial number of new HBV-derived peptides, which are presented on HLA class I allotypes that are more prevalent in regions where HBV is endemic and is a considerable health burden, could be particularly beneficial for vaccine and therapeutic development. As the HBV-derived peptides identified, particularly their anchor residues, were typically conserved across HBV genotypes A-H, it could conceivably be difficult for HBV to mutate these regions in the face of vaccine-induced T cell responses. Furthermore, in line with our previous findings (53), we found that plasma-membrane-expressed TAPBPR could be utilized to load viral-derived peptides onto HLA-A*68:02 molecules. This may have applications related to peptide-based HBV vaccines and therapeutic design.

## Data Availability

The datasets presented in this study can be found in online repositories. The names of the repository/repositories and accession number(s) can be found below: https://www.ebi.ac.uk/pride/archive/, PXD078020 (88).
