# Supplementary material for: Identification of novel HLA class I-restricted hepatitis B virus peptides and their modulation by peptide editor TAPBPR
Source: Front Immunol. 2026 Jul 31;17:1892820. doi: 10.3389/fimmu.2026.1892820 (PMC13473161; doi:10.3389/fimmu.2026.1892820)
Supplement: Supplementary file 1 [file SupplementaryFile1.docx]

Supplementary Material

# 1 Supplementary Figures and Tables

## 1.1 Supplementary Figures

**Figure S1. Validation of HeLa cells expressing a single HLA class I allotype and HBV protein.** (**A**) Wild-type (WT) or HLA-ABC^KO^ HeLa cells were left untreated or stimulated with IFN-γ. HLA class I-depletion was validated using western blot. Calnexin was used as a loading control. (**B**) Indicated HLA class I alleles were transduced into the HLA-ABC^KO^ HeLa cells. Expression on untreated cells was validated by flow cytometry. N=3, HLA-B*35:01 n=2. *p<0.05, **p<0.01, ***p<0.001, ****p<0.0001. (**C**) Indicated HBV ORFs were introduced in the single HLA class I allele HeLa cells. Expression of HBV proteins was validated by western blot by immunoblotting for FLAG. Calnexin was used as a loading control.

**Figure S2. Anchor residues of the immunopeptidome eluted from each of the cell lines.** Seqlogos (Tareen *et al*., 2020, Bioinformatics) were generated using all peptides (cellular and HBV) of 9 amino acids in length. HeLa HLA-ABC^KO^ cells transduced with one of the indicated HLA class I allotypes and HBsAg or Pol (**A**) or HBxAg or HBcAg (**B**). Peptide count used to generate each seqlogo is indicated with n. Reference seqlogos for each HLA class I allotype can be found at <http://mhcmotifatlas.org>.

```
Q8JMY6_HBSAG_HBVH1      MGAPLSTARRGMGQNLSVPNPLGFFPDHQLDPLFRANSSSPDWDFNTNKDNWPMANKVGV	60
FLAG_HBSAG              -MD-YKDDDDKMGQNLSTSNPLGFFPDHQLDPAFRANTANPDWDFNPNKDTWPDANKVGA	58
P03139_HBSAG_HBVD1      -----------MGQNLSTSNPLGFFPDHQLDPAFRANTNNPDWDFNPNKDTWPDANKVGA	49
Q69603_HBSAG_HBVE1      -MGLSWTVPLEWGKNISTTNPLGFFPDHQLDPAFRANTRNPDWDHNPNKDHWTEANKVGV	59
Q9IBI3_HBSAG_HBVG3      -MGLSWTVPLEWGKNLSASNPLGFLPDHQLDPAFRANTNNPDWDFNPKKDPWPEANKVGV	59
P31873_HBSAG_HBVA1      MGGWSAKPRKGMGTNLSVPNPLGFFPDHQLDPAFGANSNNPDWDFNPNKDHWPEANQVGV	60
P31868_HBSAG_HBVC1      MGGWSSKPRQGMGTNLSVPNPLGFFPDHQLDPAFGANSNNPDWDFNPNKDHWPEANQVGV	60
P17398_HBSAG_HBVB1      -----------MGTNLSVPNPLGFFPDHQLDPAFKANSENPDWDLNPHKDNWPDAHKVGV	49
                                    * *:*. *****:******* * **: .**** * :** *  *::**.

Q8JMY6_HBSAG_HBVH1      GGFGPGFTPPHGGLLGWSPQAQGILTTSPPDPPPASTNRRSGRKPTPVSPPLRDTHPQAM	120
FLAG_HBSAG              GAFGLGFTPPHGGLLGWSPQAQGILQTLPANPPPASTNRQSGRQPTPLSPPLRNTHPQAM	118
P03139_HBSAG_HBVD1      GAFGLGFTPPHGGLLGWSPQAQGIMQTLPANPPPASTNRQSGRQPTPLSPPLRTTHPQAM	109
Q69603_HBSAG_HBVE1      GAFGPGFTPPHGGLLGWSPQAQGMLKTLPADPPPASTNRQSGRQPTPITPPLRDTHPQAM	119
Q9IBI3_HBSAG_HBVG3      GAYGPGFTPPHGGLLGWSPQSQGTLTTLPADPPPASTNRQSGRQPTPISPPLRDSHPQAM	119
P31873_HBSAG_HBVA1      GAFGPGFTPPHGGLLGWSSQAQGTLHTVPAVPPPASTNRQTGRQPTPISPPLRDSHPQAM	120
P31868_HBSAG_HBVC1      GTFGPGFTPPHGGLLGWSPQAQGILTTVPAAPPPASTNRQSGRQPTPISPPLRDSHPQAM	120
P17398_HBSAG_HBVB1      GAFGPGFTPPHGGLLGWSPQAQGILTSVPAAPPPASTNRQSGRQPTPLSPPLRDTHPQAM	109
                        * :* ************* *:** : : *  ********::**:***::**** :*****

Q8JMY6_HBSAG_HBVH1      QWNSTQFHQALLDPRVRGLYFPAGGSSSETQNPAPTIASLTSSIFSKTGDPAMNMENITS	180
FLAG_HBSAG              QWNSTTFHQTLQDPRVRGLYFPAGGSSSGTVNPVLTTASPLSSIFSRIGDPALNMENITS	178
P03139_HBSAG_HBVD1      HWNSTTFHQTLQDPRVRGLYFPAGGSSSGTVNPVPTTTSPISSIFSRIGDPALNMENITS	169
Q69603_HBSAG_HBVE1      QWNSTTFHQALQDPRVRGLYFPAGGSSSGTVNPVPTTASLISSIFSRIGDPAPNMESITS	179
Q9IBI3_HBSAG_HBVG3      QWNSTAFHQALQNPKVRGLYFPAGGSSSGIVNPVPTIASHISSIFSRIGDPAPNMENITS	179
P31873_HBSAG_HBVA1      QWNSTAFQQALQDPRVRGLFFPAGGSSSGTVNPAPNIASHISSISSRTGDPALNMENITS	180
P31868_HBSAG_HBVC1      QWNSTTFHQALLDPRVRGLYFPAGGSSSGTVNPVPTTASPISSIFSRTGDPAPNMENTTS	180
P17398_HBSAG_HBVB1      QWNSTTFHQTLQDPRVRALYFPAGGSSSGTVSPAQNTVSAISSILSKTGDPVPNMENIAS	169
                        :**** *:*:* :*:**.*:********   .*. . .*  *** *: ***. ***. :*

Q8JMY6_HBSAG_HBVH1      GLLRPLLVLQAVCFLLTKILTIPQSLDSWWTSLNFLGVPPGCPGQNSQSPISNHLPTSCP	240
FLAG_HBSAG              GFLGPLLVLQAGFFLLTRILTIPQSLDSWWTSLNFLGGTTVCLGQNSQSPTSNHSPTSCP	238
P03139_HBSAG_HBVD1      GFLGPLLVLQAGFFLLTRILTIPQSLDSWWTSLNFLGGTTVCLGQNSQSPISNHSPTSCP	229
Q69603_HBSAG_HBVE1      GFLGPLLVLQAGFFLLTKILTIPQSLDSWWTSLNFLGGAPVCLGQNSQSPTSNHSPTSCP	239
Q9IBI3_HBSAG_HBVG3      GFLGPLLVLQAGFFLLTRILTIPQSLDSWWTSLNFLGGVPVCPGLNSQSPTSNHSPISCP	239
P31873_HBSAG_HBVA1      GFLGPLLVLQAGFFLLTRILTIPQSLDSWWTSLNFLGGSPVCLGQNSQSPTSNHSPTSCP	240
P31868_HBSAG_HBVC1      GFLGPLLVLQAGFFLLTRILTIPQSLDSWWTSLNFLGGAPTCPGQNSQSPTSNHSPTSCP	240
P17398_HBSAG_HBVB1      GLLGPLLVLQAGFFLLTKILTIPQSLDSWWTSLNFLGGTPVCLGQNSQSQISSHSPTCCP	229
                        *:* *******  ****:*******************    * * ****  *.* * .**

Q8JMY6_HBSAG_HBVH1      PTCPGYRWMCLRRFIIFLFILLLCLIFLLVLLDYQGMLPVCPLLPGSTTTSTGPCKTCTT	300
FLAG_HBSAG              PTCPGYRWMCLRRFIIFLFILLLCLIFLLVLLDYQGMLPVCPLIPGSSTTSTGPCRTCMT	298
P03139_HBSAG_HBVD1      PTCPGYRWMCLRRFIIFLFILLLCLIFLLVLLDYQGMLPVCPLIPGSSTTSTGSCRTCTT	289
Q69603_HBSAG_HBVE1      PICPGYRWMCLRRFIIFLFILLLCLIFLLVLLDYQGMLPVCPLIPGSSTTSTGPCRTCMT	299
Q9IBI3_HBSAG_HBVG3      PTCPGYRWMCLRRFIIFLFILLLCLIFLLVLLDYQGMLPVCPLIPGSSTTSTGPCKTCTT	299
P31873_HBSAG_HBVA1      PICPGYRWMCLRRFIIFLFILLLCLIFLLVLLDYQGMLPVCPLIPGSTTTSTGPCKTCTT	300
P31868_HBSAG_HBVC1      PICPGYRWMCLRRFIIFLFILLLCLIFLLVLLDYQGMLPVCPLLPGTSTTSTGPCKTCTT	300
P17398_HBSAG_HBVB1      PICPGYRWMCLRRFIIFLCILLLCLIFLLVLLDYQGMLPVCPLIPGSSTTSTGPCKTCTT	289
                        * **************** ************************:**::***** *:** *

Q8JMY6_HBSAG_HBVH1      LAQGTSMFPSCCCTKPSDGNCTCIPIPSSWAFGKYLWEWASARFSWLSLLVQFVQWCVGL	360
FLAG_HBSAG              TAQGTSMYPSCCCTKPSDGNCTCIPIPSSWAFGKFLWEWASARFSWLSLLVPFVQWFVGL	358
P03139_HBSAG_HBVD1      PAQGISMYPSCCCTKPSDGNCTCIPIPSSWAFGKFLWEWASARFSWLSLLVPFVQWFVGL	349
Q69603_HBSAG_HBVE1      LAQGTSMFPSCCCSKPSDGNCTCIPIPSSWAFGKFLWEWASARFSWLSLLVPFVQWFAGL	359
Q9IBI3_HBSAG_HBVG3      PAQGNSMYPSCCCTKPSDGNCTCIPIPSSWAFAKYLWEWASVRFSWLSLLVPFVQWFVGL	359
P31873_HBSAG_HBVA1      PAQGNSMFPSCCCTKPTDGNCTCIPIPSSWAFAKYLWEWASVRFSWLSLLVPFVQWFVGL	360
P31868_HBSAG_HBVC1      PAQGTSMFPSCCCTKPSDGNCTCIPIPSSWAFARFLWEWASVRFSWLSLLVPFVQWFAGL	360
P17398_HBSAG_HBVB1      PAQGTSMFPSCCCTKPMDGNCTCIPIPSSWAFAKYLWEWASVRFSWLSLLVPFVQWFVGL	349
                         *** **:*****:** ***************.::******.********* **** .**

Q8JMY6_HBSAG_HBVH1      SPTVWLLVIWMIWYWGPNLCSILSPFIPLLPIFCYLWASI	400
FLAG_HBSAG              SPTVWLSVIWMMWYWGPSLYSILSPFLPLLPIFFCLWVYI	398
P03139_HBSAG_HBVD1      SPIVWLSVIWMMWYWGPSLYSILSPFLPLLPIFFCLWAYI	389
Q69603_HBSAG_HBVE1      SPTVWLSVIWMMWYWGPSLYDILSPFIPLLPIFFCLWVYI	399
Q9IBI3_HBSAG_HBVG3      SPTVWLSAIWMMWYWGPNLYNILSPFIPLLPIFFCLWVYI	399
P31873_HBSAG_HBVA1      SPTVWLSVIWMMWYWGPSLYNILSPFIPLLPIFFCLWVYI	400
P31868_HBSAG_HBVC1      SPTVWLSVIWMMWYWGPSLYNILSPFLPLLPIFFCLWVYI	400
P17398_HBSAG_HBVB1      SPTVWLSVIWMMWYWGPSLYNILSPFMPLLPIFFCLWVYI	389
                        ** *** .***:*****.* .*****:******  **. *
```

FLAG tag

HLA-restriction:

A0201 (overlap with another peptide)

B0801

B3501 (overlap with other peptides)

*C0602* *(overlap with another peptide)*

C1203 (overlap with peptides)

**Figure S3. All HBsAg-derived peptides have at least one anchor residue conserved across HBV genotypes A-H**. HBsAg sequences obtained from Uniprot (accession numbers indicated) were aligned using Clustal Omega (Madeira *et al*., 2024, Europe PMC) with the FLAG-tagged sequence transduced into HeLa cells. Epitopes detected by immunopeptidomics are highlighted. Bold = expected anchor residue. Bold, coloured text without background = mismatch between genotypes but tolerated anchor residue. Underlined = unexpected anchor residue (**Figure S2** and <http://mhcmotifatlas.org>).

```
Q05486_DPOL_HBVF1      -----------------------MPLSYPHFRKLLLLDD--EAGPLEEELPRLADEDLNR	35
Q8JMY7_DPOL_HBVH1      -----------------------MPLSYQHFRRLLLLDN--EAGPLEEELPRLADEDLNH	35
P17394_DPOL_HBVB1      -----------------------MPLSYQHFRKLLLLDD--EAGPLEEELPRLADEGLNH	35
P03158_DPOL_HBVA2      -----------------------MPLSYQHFRKLLLLDDGTEAGPLEEELPRLADADLNR	37
P0C688_DPOL_HBVC1      -----------------------MPLSYQHFRKLLLLDD--EAGPLEEELPRLADEGLNR	35
3xFLAG_Pol             MDYKDHDGDYKDHDIDYKDDDDKMPLSYQHFRRLLLLDD--EAGPLEEELPRLADEGLNR	58
P03155_DPOL_HBVD1      -----------------------MPLSYQRFRRLLLLDD--EAGPLEEELPRLADEDLNR	35
Q69602_DPOL_HBVE1      -----------------------MPLSYQHFRRILLLDE--EAGPLEEELPRLADEDLNR	35
Q8QZQ2_DPOL_HBVG2      -----------------------MPLSYQHFRRLLLLDE--EAGPLEEELPRLADEDLNR	35
                                              ***** :**::****:  ************** .**:

Q05486_DPOL_HBVF1      RVAADLNLQLPNVSIPWTHKVGNFTGLYSSTVPAFNPNWSTPSFPDIHLHQDLISKCEQF	95
Q8JMY7_DPOL_HBVH1      RVAEDLNLQLPNVSIPWTHKVGNFTGLYSSTIPVFNPDWLTPSFPDIHLHQDLIQKCEQF	95
P17394_DPOL_HBVB1      RVAEDLNLGNPNVSIPWTHKVGNFTGLYSSTVPVFNPEWQTPSFPDIHLQEDIVDRCKQF	95
P03158_DPOL_HBVA2      RVAEDLNLGNLNVSIPWTHKVGNFTGLYSSTVPIFNPEWQTPSFPKIHLQEDIINRCQQF	97
P0C688_DPOL_HBVC1      RVAEDLNLGNLNVSIPWTHKVGNFTGLYSSTVPVFNPEWQTPSFPNIHLQEDIINRCQQY	95
3xFLAG_Pol             RVAEDLNLGNLNVSIPWTHKVGNFTGLYSSTVPVFNPHWKTPSFPNIHLHQDIIKKCEQF	118
P03155_DPOL_HBVD1      RVAEDLNLGNLNVSIPWTHKVGNFTGLYSSTVPVFNPHWKPPSFPNIHLHQDIIKKCEQF	95
Q69602_DPOL_HBVE1      RVAEDLNLQLPNVSIPWTHKVGNFTGLYSSTIPVFNPNWKTPSFPDIHLHQDIINKCEQF	95
Q8QZQ2_DPOL_HBVG2      RVAEDLHLQLPNVSIPWTHKVGNFTGLYSSTIPVFNPDWQTPSFPNIHLHQDIITKCEQF	95
                       *** **:*   ********************:* ***.*  ****.***::*:: :*:*:

Q05486_DPOL_HBVF1      VGPLTKNELRRLKLVMPARFYPKVTKYFPMDKGIKPYYPEHAVNHYFKTRHYLHTLWKAG	155
Q8JMY7_DPOL_HBVH1      VGPLTTNERRRLKLIMPARFYPKVTKYFPLDKGIKPYYPENVVNHYFKTRHYLHTLWKAG	155
P17394_DPOL_HBVB1      VGPLTVNENRRLKLIMPARFYPNVTKYLPLDKGIKPYYPEHVVNHYFQTRHYLHTLWKAG	155
P03158_DPOL_HBVA2      VGPLTVNEKRRLKLIMPARFYPTHTKYLPLDKGIKPYYPDQVVNHYFQTRHYLHTLWKAG	157
P0C688_DPOL_HBVC1      VGPLTVNEKRRLKLIMPARFYPNLTKYLPLDKGIKPYYPEHAVNHYFKTRHYLHTLWKAG	155
3xFLAG_Pol             VGPLTVNEKRRLQLIMPARFYPKVTKYLPLDKGIKPYYPEHLVNHYFQTRHYLHTLWKAG	178
P03155_DPOL_HBVD1      VGPLTVNEKRRLKLIMPARFYPNFTKYLPLDKGIKPYYPEHLVNHYFQTRHYLHTLWKAG	155
Q69602_DPOL_HBVE1      VGPLTVNEKRRLNLVMPARFFPISTKYLPLEKGIKPYYPDNVVNHYFQTRHYLHTLWKAG	155
Q8QZQ2_DPOL_HBVG2      VGPLTVNEKRRLKLVMPARFFPNSTKYLPLDKGIKPYYPENVVNHYFQTRHYLHTLWKAG	155
                       ***** ** ***:*:*****:*  ***:*::********:: *****:************

Q05486_DPOL_HBVF1      ILYKRESTRSASFCGSPYSWEQELQHGSTSLNDTKRHGTESLCAQSSGILSRPSAGSAIQ	215
Q8JMY7_DPOL_HBVH1      ILYKRESTHSASFCGSPYSWEQELQHGSTSLNGEKGHGTEPFCAQSSGILSRPPVGSTIQ	215
P17394_DPOL_HBVB1      ILYKRESTHSASFCGSPYSWEQDLQHGRLVFQTSKRHGDKSFCPQSPGILPRSSVGPCIQ	215
P03158_DPOL_HBVA2      ILYKRETTRSASFCGSPYSWEQELQH-------SQRHGDESFCSQPSGIPSRSSVGPCIR	210
P0C688_DPOL_HBVC1      ILYKRETTRSASFCGSPYSWEQELQHGRLVFQTSTRHGDESFCSQSSGILSRSPVGPCIR	215
3xFLAG_Pol             ILYKRETTHSASFCGSPYSWEQDLQHGA-----------ESFHQQSSGILSRPPVGSSLQ	227
P03155_DPOL_HBVD1      VLYKRVSTHSASFCGSPYSWEQELQHGA-----------ESFHQQSSGILSRPPVGSSLQ	204
Q69602_DPOL_HBVE1      HLYKRETTRSASFCGSPYSWEQELHHGAFLD-GPSRMGEEYFHHQSSGIFSRPPVGSSIQ	214
Q8QZQ2_DPOL_HBVG2      ILYKRETSRSASFCGSPYTWEQDLQHGAFLD-GPSRVGKEPFHQQSSRIPSRSPVGPSIQ	214
                        **** :::*********:***:*:*             : :  *   *  *  .*  ::

Q05486_DPOL_HBVF1      SKFQQSRLGLQHKQGQLANGKQGRSGRLRSRVHTPTRWPAGVEPSSTRCVNNLASRSASC	275
Q8JMY7_DPOL_HBVH1      SKFQQSRLGLQHKQGQLANGKQGRSGRLRSRVHTPTRWPSGVEPSGTGHSDNLATRSTSC	275
P17394_DPOL_HBVB1      SQLRKSRLGPQPTQGQLAGRPQGGSGSIRARIHPSPWGTVGVEPSGSGHTHICASSSSSC	275
P03158_DPOL_HBVA2      SQLNKSRLGLQPHQGPLASSQPGRSGSIRARAHPSTRRYFGVEPSGSGHIDHSVNNSSSC	270
P0C688_DPOL_HBVC1      SQLKQSRLGLQPQQGSLARGKSGRSGSIWARVHSTTRRSFGVEPSGSGHIDNSASSASSC	275
3xFLAG_Pol             SKHRKSRLGLQSQQGHLARRQQGRSWSIRAGFHPTARRPFGVEPSGSGHTTNFASKSASC	287
P03155_DPOL_HBVD1      SKHQQSRLGLQSQQGHLARRQQGRSWSIRARVHPTARRPFGVEPSGSGHNANLASKSASC	264
Q69602_DPOL_HBVE1      SKHQKSRLGPQSQQRPLDGSQQGRSGSLRAGVHSPTRRPFGVEPSGSRHAKNIASRPASC	274
Q8QZQ2_DPOL_HBVG2      SKYQQSRLGLQSQKGPLARGQQGRSWSLWTRVHPSTRRPFGVEPSVSGHTNNFASRSASC	274
                       *: .:**** *  :  *     * *  : :  *       ***** :      ..  :**

Q05486_DPOL_HBVF1      FHQSAVREKANPSLSTSKRHTSTGNAVELNPVPPSSVGSQGKGSVLPCWWLQFRDTEPCS	335
Q8JMY7_DPOL_HBVH1      FHQSEVRKKANPSLSTSKGHTSTGHAVELNTVPPSTVGSESKGSVFSCWWLQFRNTEPCS	335
P17394_DPOL_HBVB1      LHQSAVRTAAYSPISTSKGHSSSGHAVELHHFPPNSSRSQSQGSVLSCWWLQFRNSKPCS	335
P03158_DPOL_HBVA2      LHQSAVRKAAYSHLSTSKRQSSSGHAVEFHCLAPSSAGSQSQGSVSSCWWLQFRNSKPCS	330
P0C688_DPOL_HBVC1      LYQSAVRKTAYSHLSTSKRQSSSGHAVELHNIPPSCARSQSEGPISSCWWLQFRNSEPCS	335
3xFLAG_Pol             LHQSPVRKAAYPAVSTFEKHSSSGHAVEFHNLPPNSARSQSERPVFPCWWLQFRNSKPCS	347
P03155_DPOL_HBVD1      LYQSPVRTAAYPAVSTSENHSSSGHALELHNLPPNSARSQSERPVFPCWWLQFRDSKPCS	324
Q69602_DPOL_HBVE1      LHQSAVRKAAYPNHSTFERHSSSGHAVELHNISSSSAGSQSKRPVFSCWWLQFRNSEPCS	334
Q8QZQ2_DPOL_HBVG2      LHQSSVREAAYSHLSTTKRQSSSGHAVELYSIPPSSTKSQSQGPVFSCWWLQFRDSEPCS	334
                       ::** **  *    ** : ::*:*:*:*:  .  .   *:.:  :  *******:::***
```

```
Q05486_DPOL_HBVF1      DYCLSHIINLLEDWGPCYEHGQHYIRTPRTPARVTGGVFLVDKNPHNTTESRLVVDFSQF	395
Q8JMY7_DPOL_HBVH1      DYCLSHIINLLEDWGPCYEHGEHHIRTPKTPSRVTGGVFLVDKNPHNTTESRLVVDFSQF	395
P17394_DPOL_HBVB1      EYCLSHIVNLIEDWGPCAEHGEHRIRTPRTPARVTGGVFLVDKNPHNTTESRLVVDFSQF	395
P03158_DPOL_HBVA2      EYCLSHLVNLREDWGPCDDHGEHHIRIPRTPARVTGGVFLVDKNPHNTAESRLVVDFSQF	390
P0C688_DPOL_HBVC1      DYCLTHIVNLLEDWGPCTEHGEHNIRIPRTPARVTGGVFLVDKNPHNTTESRLVVDFSQF	395
3xFLAG_Pol             DYCLSLIVNLLEDWGPCAEHGEHHIRIPRTPSRVTGGVFLVDKNPHNTAESRLVVDFSQF	407
P03155_DPOL_HBVD1      DYYLSHIVNLLEDWGPCAEHGEHHIRIPRTPARVTGGVFLVDKNPHNTAESRLVVDFSQF	384
Q69602_DPOL_HBVE1      DYCLTHLVNLLEDWGPCTEHGKHHIRIPRTPARVTGGVFLVDKNPHNTAESRLVVDFSQF	394
Q8QZQ2_DPOL_HBVG2      DYCLSHLVNLLQDWGPCTEHGEYHIRIPRTPARVTGGVFLVDKNPHNTAESRLVVDFSQF	394
                       :* *: ::** :***** :**:: ** *:**:****************:***********

Q05486_DPOL_HBVF1      SRGTTRVSWPKFAVPNLQSLTNLLSSNLSWLSLDVSAAFYHLPLHPAAMPHLLVGSSGLS	455
Q8JMY7_DPOL_HBVH1      SRGTTRVSWPKFAVPNLQSLTNLLSSNLSWLSLDVSAAFYHLPLHPAAMPHLLVGSSGLS	455
P17394_DPOL_HBVB1      SRGNTRVSWPKFAVPNLQSLTNLLSSNLSWLSLDVSAAFYHLPLHPAAMPHLLVGSSGLS	455
P03158_DPOL_HBVA2      SRGITRVSWPKFAVPNLQSLTNLLSSNLSWLSLDVSAAFYHIPLHPAAMPHLLIGSSGLS	450
P0C688_DPOL_HBVC1      SRGSTHVSWPKFAVPNLQSLTNLLSSNLSWLSLDVSAAFYHIPLHPAAMPHLLVGSSGLP	455
3xFLAG_Pol             SRGNYRVSWPKFAVPNLQSLTNLLSSNLSWLSLDVSAAFYHLPLHPAAMPHLLVGSSGLS	467
P03155_DPOL_HBVD1      SRGNYRVSWPKFAVPNLQSLTNLLSSNLSWLSLDVSAAFYHLPLHPAAMPHLLVGSSGLS	444
Q69602_DPOL_HBVE1      SRGSSRVSWPKFAVPNLQSLTNLLSSNLSWLSLDVSAAFYHLPLHPAAMPHLLVGSSGLS	454
Q8QZQ2_DPOL_HBVG2      SRGSARVSWPKFAVPNLQSLTNLLSSNLSWLSLDVSAAFYHIPLHPAAMPHLLVGSSGLS	454
                       ***  :***********************************:***********:*****

Q05486_DPOL_HBVF1      RYVARLSSTSRIHDHQHGTLQNLHNSCTRNLYVSLLLLFQTLGRKLHLYSHPIILGFRKI	515
Q8JMY7_DPOL_HBVH1      RYVARVSSTSRIYNHQHGTLQNLHHSCSRNLYVSLLLLYQTFGRKLHLYSHPIILGFRKI	515
P17394_DPOL_HBVB1      RYVARLSSNSRIINHQHGTMQDLHNSCSRNLYVSLMLLYKTYGWKLHLYSHPIILGFRKI	515
P03158_DPOL_HBVA2      RYVARLSSNSRINNNQYGTMQNLHDSCSRQLFVSLMLLYKTYGWKLHLYSHPIVLGFRKI	510
P0C688_DPOL_HBVC1      RYVARLSSTSRNINYQHGTMQDLHDSCSRNLYVSLLLLYKTFGRKLHLYSHPIILGFRKI	515
3xFLAG_Pol             RYVARLSSNSRILNNQHGTMPDLHDYCSRNLYVSLLLLYQTFGRKLHLYSHPIILGFRKI	527
P03155_DPOL_HBVD1      RYVARLSSNSRIINHQHGILQNLHDSCSRNLYVSLLLLYKTFGWKLHLYSHPIILGFRKI	504
Q69602_DPOL_HBVE1      RYVARLSSNSRIINHQHGTLPNLHDSCSRNLYVSLMLLFKTFGRKLHLYSHPIIMGFRKI	514
Q8QZQ2_DPOL_HBVG2      RYVARLSSDSRILDHQYGTLQNLHDSCSRQLYVSLMLLYKTFGRKLHLYSHPIILGFRKI	514
                       *****:** **  : *:* : :**. *:*:*:***:**::* * *********::*****

Q05486_DPOL_HBVF1      PMGVGLSPFLLAQFTSAICSVVRRAFPHCLAFSYMDDLVLGAKSVQHLESLYTAVTNFLL	575
Q8JMY7_DPOL_HBVH1      PMGVGLSPFLLAQFTSAICSVVRRAFPHCLAFSYMDDLVLGAKSVQHLESLYTAVTNFLL	575
P17394_DPOL_HBVB1      PMGVGLSPFLLAQFTSAICSVVRRAFPHCLAFSYMDDVVLGAKSVQHLESLYAAVTNFLL	575
P03158_DPOL_HBVA2      PMGVGLSPFLLAQFTSAICSVVRRAFPHCLAFSYMDDVVLGAKSVQHRESLYTAVTNFLL	570
P0C688_DPOL_HBVC1      PMGVGLSPFLLAQFTSAICSVVRRAFPHCLAFSYMDDVVLGAKSVQHLESLFTSITNFLL	575
3xFLAG_Pol             PMGVGLSPFLLAQFTSAICSVVRRAFPHCLAFSYMDDVVLGAKSVQHLESLFTAVTNFLL	587
P03155_DPOL_HBVD1      PMGVGLSPFLLAQFTSAICSVVRRAFPHCLAFSYMDDVVLGAKSVQHLESLFTAVTNFLL	564
Q69602_DPOL_HBVE1      PMGVGLSPFLLAQFTSAICSVVRRAFPHCLAFSYMDDVVLGAKSVRHLESLYTSVTNFLL	574
Q8QZQ2_DPOL_HBVG2      PMGVGLSPFLLAQFTSAICSVVRRAFPHCLAFSYMDDVVLGAKSVQHLESLYTAVTNFLL	574
                       *************************************:*******:* ***::::*****

Q05486_DPOL_HBVF1      SVGIHLNTSKTKRWGYSLHFMGYVIGSWGSLPQDHIVHKIKECFRKLPVNRPIDWKVCQR	635
Q8JMY7_DPOL_HBVH1      SVGIHLNTAKTKWWGYSLHFMGYIIGSWGTLPQEHIVHKIKDCFRKLPVNRPIDWKVCQR	635
P17394_DPOL_HBVB1      SLGIHLNPNKTKRWGYSLNFMGYVIGSWGTWPQDHIVQNFKLCFRKLPVNRPIDWKVCQR	635
P03158_DPOL_HBVA2      SLGIHLNPNKTKRWGYSLNFMGYIIGSWGTLPQDHIVQKIKHCFRKLPVNRPIDWKVCQR	630
P0C688_DPOL_HBVC1      SLGIHLNPHKTKRWGYSLNFMGYVIGSWGTLPQEHIVLKIKQCFRKLPVNRPIDWKVCQR	635
3xFLAG_Pol             SLGIHLNPNKTKRWGYSLNFMGYVIGCYGSLPQEHIIQKIKECFRKLPINRPIDWKVCQR	647
P03155_DPOL_HBVD1      SLGIHLNPNKTKRWGYSLNFMGYVIGCWGSLPQDHIIHKIKECFRKLPVHRPIDWKVCQR	624
Q69602_DPOL_HBVE1      SLGIHLNPNKTKRWGYSLNFMGYVIGSWGSLPQEHIIQKIKHCFGKLPVNRPIDWKVCQG	634
Q8QZQ2_DPOL_HBVG2      SLGIHLNPNKTKRWGYSLNFMGYVIGSWGTLPQEHITQKIKQCFRKLPVNRPIDWKVCQR	634
                       *:*****  *** *****:****:**.:*: **:**  ::* ** ***::*********

Q05486_DPOL_HBVF1      IVGLLGFAAPFTQCGYPALMPLYACITAKQAFVFSPTYKAFLCKQYMNLYPVARQRPGLC	695
Q8JMY7_DPOL_HBVH1      IVGLLGFAAPFTQCGYPALMPLYACITAKQAFVFSPTYKAFLCKQYMNLYPVARQRPGLC	695
P17394_DPOL_HBVB1      IVGLLGFAAPFTQCGYPALMPLYACIQAKQAFTFSPTYKAFLSKQYMTLYPVARQRPGLC	695
P03158_DPOL_HBVA2      IVGLLGFAAPFTQCGYPALMPLYACIQAKQAFTFSPTYKAFLSKQYMNLYPVARQRPGLC	690
P0C688_DPOL_HBVC1      IVGLLGFAAPFTQCGYPALMPLYACIQSKQAFTFSPTYKAFLCKQYLHLYPVARQRSGLC	695
3xFLAG_Pol             IVGLLGFAAPFTQCGYPALMPLYACIQSKQAFTFSPTYKAFLCKQYLNLYPVARQRPGLC	707
P03155_DPOL_HBVD1      IVGLLGFAAPFTQCGYPALMPLYACIQSKQAFTFSPTYKAFLCKQYLNLYPVAEQRPGLC	684
Q69602_DPOL_HBVE1      IVGLLGFAAPFTQCGYPALMPLYTCIQSKQAFTFSPTYKAFLCKQYLNLYPVARQRPGLC	694
Q8QZQ2_DPOL_HBVG2      ITGLLGFAAPFTQCGYPALMPLYACIQAKQAFTFSPTYKAFLCKQYMNLYPVARQRPGLC	694
                       *.*********************:** :****.*********.***: *****.** ***
```

```
Q05486_DPOL_HBVF1      QVFADATPTGWGLAIGHQRMRGTFVAPLPIHTAELLAACFARSRSGATLIGTDNSVVLSR	755
Q8JMY7_DPOL_HBVH1      QVFADATPTGWGLAIGHQRMRGTFVAPLPIHTAELLAACFARSRSGADIIGTDNSVVLSR	755
P17394_DPOL_HBVB1      QVFADATPTGWGLAIGHQRMRGTFVSPLPIHTAELLAACFARSRSGANLIGTDNSVVLSR	755
P03158_DPOL_HBVA2      QVFADATPTGWGLAIGHQRMRGTFVAPLPIHTAELLAACFARSRSGAKLIGTDNSVVLSR	750
P0C688_DPOL_HBVC1      QVFADATPTGWGLAIGQSGMRGTFVAPLPIHTAELLAACFARSRSGAKLIGTDNSVVLSR	755
3xFLAG_Pol             QVFADATPTGWGLVMGHQRMRGTFSAPLPIHTAELLAACFARSRSGANIIGTDNSVVLSR	767
P03155_DPOL_HBVD1      QVFADATPTGWGLVMGHQRMRGTFLAPLPIHTAELLAACFARSRSGANILGTDNSVVLSR	744
Q69602_DPOL_HBVE1      QVFADATPTGWGLAIGIQRMRGTFVAPLPIHTAELLAACFARSRSGAKLIGTDNSVVLSR	754
Q8QZQ2_DPOL_HBVG2      QVFADATPTGWGLAIGHQRMRGTFVAPLPIHTAELLAACFARSRSGAKLIGTDNSVVLSR	754
                       *************.:* . ***** :********************* ::**********

Q05486_DPOL_HBVF1      KYTSFPWLLGCAANWILRGTSFVYVPSALNPADDPSRGRLGLYRPLLRLPFQPTTGRTSL	815
Q8JMY7_DPOL_HBVH1      KYTSFPWLLGCAANWILRGTSFVYVPSALNPADDPSRGRLGLCRPLLRLPFRPTTGRTSL	815
P17394_DPOL_HBVB1      KYTSFPWLLGCAANWILRGTSFVYVPSALNPADDPSRGRLGLYRPLLRLPYRPTTGRTSL	815
P03158_DPOL_HBVA2      KYTSFPWLLGCTANWILRGTSFVYVPSALNPADDPSRGRLGLSRPLLRLPFQPTTGRTSL	810
P0C688_DPOL_HBVC1      KYTSFPWLLGCAANWILRGTSFVYVPSALNPADDPSRGRLGLYRPLLHLPFRPTTGRASL	815
3xFLAG_Pol             KYTSFPWLLGCAANWILRGTSFVYVPSALNPADDPSRGRLGLSRPLLRLPFRPTTGRTSL	827
P03155_DPOL_HBVD1      KYTSFP------------------------------------------------------	749
Q69602_DPOL_HBVE1      KYTSFPWLLGCAANWILRGTSFVYVPSALNPADDPSRGRLGIFRPLLRLRFRPTTGRTSL	814
Q8QZQ2_DPOL_HBVG2      KYTSFPWLLGCAANWILRGTSFVYVPSALNPADDPSRGRLGLCRPLLRLPFLPTTGRTSL	814
                       ******

Q05486_DPOL_HBVF1      YADSPSVPSHLPDRVHFASPLHVAWRPP	842
Q8JMY7_DPOL_HBVH1      YADSPPVPSHLPARVHFASPLHVAWRPP	842
P17394_DPOL_HBVB1      YADSPSVPSHLPDRVHFASPLHVAWRPP	842
P03158_DPOL_HBVA2      YAVSPSVPSHLPVRVHFASPLHVAWRPP	837
P0C688_DPOL_HBVC1      YAVSPSVPSHLPVRVHFASPLHVAWRPP	842
3xFLAG_Pol             YADSPSVPSHLPDRVHFASPLHVAWRPP	854
P03155_DPOL_HBVD1      ----------------------------	749
Q69602_DPOL_HBVE1      YAVSPSVPSHLPDRVHFASPLHVAWRPP	841
Q8QZQ2_DPOL_HBVG2      YAVSPSVPSHLPDRVHFASPLHVTWKPP	841

3x FLAG

HLA-restriction:
A0201
B0801 (overlap with another peptide)
B3501 (overlap with another peptide)
C0602 (overlap with another peptide)
A6802 (overlap with another peptide)
B1503 (overlap with another peptide)
C1203 (overlap with another peptide)
```

**Figure S4. Most of Pol-derived peptides have anchor residues conserved across all or most HBV genotypes A-H**. HBV Pol sequences obtained from Uniprot (accession numbers indicated) were aligned using Clustal Omega (Madeira *et al*., 2024, Europe PMC) with the FLAG-tagged sequence transduced into HeLa cells. Epitopes detected by immunopeptidomics are highlighted. Bold = expected anchor residue. Bold, coloured text without background = mismatch between genotypes but tolerated anchor residue. Underlined = unexpected anchor residue (**Figure S2** and <http://mhcmotifatlas.org>).

```
Q81102_CAPSD_HBVC1       ------------MDIDTYKEFGASVELLSFLPSDFFPSIRDLLDTAFALHREALESPEHC	48
P0C6I4_CAPSD_HBVF1       ------------MDIDPYKEFGASVELLSFLPSDFFPSVRDLLDTASALYRDALESPEHC	48
P0C6I6_CAPSD_HBVH3       ------------MDIDPYKEFGASVELLSFLPSDFFPSVRDLLDTASALYRDALESPEHC	48
P03147_CAPSD_HBVD1       ------------MDIDPYKEFGATVELLSFLPSDFFPSVRDLLDTAAALYRDALESPEHC	48
Q89531_Q89531_HBVE1      ------------MDIDPYKEFGATVELLSFLPSDFFPSVRDLLDTASALYRDALESPEHC	48
P29178_CAPSD_HBVG1       MDRTTLPYGLFGLDIDPYKEFGATVELLSFLPSDFFPSVRDLLDTASALYRESLESSDHC	60
P17391_CAPSD_HBVB1       ------------MDIDPYKEFGATVELLSFLPSDFFPSVRDLLDTVSALYREALKSPEHC	48
HBcAg-FLAG               ------------MDIDPYKEFGATVELLSFLPSDFFPSVRDLLDTASALYREALESPEHC	48
P03149_CAPSD_HBVA2       ------------MDIDPYKEFGATVELLSFLPSDFFPSVRDLLDTASALYREALESPEHC	48
                                     :*** ******:**************:******. **:*::*:* :**

Q81102_CAPSD_HBVC1       SPHHTALRQAIVCWGELMNLATWVGSNLEDPASRELVVSYVNVNMGLKIRQLLWFHISCL	108
P0C6I4_CAPSD_HBVF1       TPNHTALRQAILCWGELMTLASWVGNNLEDPAARDLVVNYVNTNMGLKIRQLLWFHISCL	108
P0C6I6_CAPSD_HBVH3       TPNHTALRQAILCWGELMTLASWVGNNLEDPAARDLVVNYVNTNMGLKIRQLLWFHISCL	108
P03147_CAPSD_HBVD1       SPHHTALRQAILCWGDLMTLATWVGTNLEDPASRDLVVSYVNTNVGLKFRQLLWFHISCL	108
Q89531_Q89531_HBVE1      SPHHTALRQAILCWGELMTLATWVGVNLEDPASRDLVVSYVNTNMGLKFRQLLWFHISCL	108
P29178_CAPSD_HBVG1       SPHHTALRQAILCWVELMTLATWVGNNLEDPASRDLVVNYVNTNMGLKIRQLLWFHISCL	120
P17391_CAPSD_HBVB1       SPHHTALRQAILCWGELMTLATWVGNNLEDPASRDLVVNYVNTNMGLKIRQLWWFHISCL	108
HBcAg-FLAG               SPHHTALRQAILCWGELMTLATWVGNNLEDPASRDLVVNYVNTNMGLKIRQLLWFHISCL	108
P03149_CAPSD_HBVA2       SPHHTALRQAILCWGELMTLATWVGNNLQDPASRDLVVNYVNTNMGLKIRQLLWFHISCL	108
                         :*:********:** :**.**:*** **:***:*:***.***.*:***:*** *******

Q81102_CAPSD_HBVC1       TFGRETVLEYLVSVGVWIRTPQAYRPPNAPILSTLPETTVVRR--RGRSPRRRTPSPRRR	166
P0C6I4_CAPSD_HBVF1       TFGRETVLEYLVSFGVWIRTPPAYRPPNAPILSTLPETTVVRR--RGRSPRRRTPSPRRR	166
P0C6I6_CAPSD_HBVH3       TFGRDTVLEYLVSFGVWIRTPPAYRPPNAPILSTLPETTVVRQ--RGRAPRRRTPSPRRR	166
P03147_CAPSD_HBVD1       TFGRETVLEYLVSFGVWIRTPPAYRPPNAPILSTLPETTVVRR--RGRSPRRRTPSPRRR	166
Q89531_Q89531_HBVE1      TFGRETVIEYLVSFGVWIRTPPAYRPPNAPILSTLPENTVVRR--RGRSPRRRTPSPRRR	166
P29178_CAPSD_HBVG1       TFGRETVLEYLVSFGVWIRTPPAYRPPNAPILSTLPETTVVRR--RGRSPRRRTPSPRRR	178
P17391_CAPSD_HBVB1       TFGRETVLEYLVSFGVWIRTPPAYRPPNAPILSTLPETTVVRR--RGRSPRRRTPSPRRR	166
HBcAg-FLAG               TFGRETVLEYLVSFGVWIRTPPAYRPPNAPILSTLPETTVVRRRDRGRSPRRRTPSPRRR	168
P03149_CAPSD_HBVA2       TFGRETVLEYLVSFGVWIRTPPAYRPPNAPILSTLPETTVVRRRDRGRSPRRRTPSPRRR	168
                         ****:**:*****.******* ***************.****:  ***:***********

Q81102_CAPSD_HBVC1       RSKSPRRRRSQSRESQC--------	183
P0C6I4_CAPSD_HBVF1       RSQSPRRRRSQSPASQC--------	183
P0C6I6_CAPSD_HBVH3       RSQSPRRRRSQSPASQC--------	183
P03147_CAPSD_HBVD1       RSQSPRRRRSQSRESQC--------	183
Q89531_Q89531_HBVE1      RSQSPRRRRSQSPASQC--------	183
P29178_CAPSD_HBVG1       RSPSPRRRRSQSRESQC--------	195
P17391_CAPSD_HBVB1       RSQSPRRRRSQSRESQC--------	183
HBcAg-FLAG               RSQSPRRRRSQSRESQCDYKDDDDK	193
P03149_CAPSD_HBVA2       RSQSPRRRRSQSRESQC--------	185
                         ** *********  ***

FLAG tag

HLA-restriction:
A0201
B0801
C0602
B1503
C1203
```

**Figure S5. All five HBcAg-derived peptides show conserved anchor residues across HBV genotypes A-H**. HBV capsid sequences obtained from Uniprot (accession numbers indicated) were aligned using Clustal Omega (Madeira *et al*., 2024, Europe PMC) with the FLAG-tagged sequence transduced into HeLa cells. Epitopes detected by immunopeptidomics are highlighted. Bold = expected anchor residue. Bold, coloured text without background = mismatch between genotypes but tolerated anchor residue (**Figure S2** and <http://mhcmotifatlas.org>).

**Figure S6. Validation of TAPBPR^KO^ HeLa cells expressing a single HBV protein.** TAPBPR was depleted from HeLa cells. (**A**) Validation of TAPBPR^KO^ HeLa cells treated with IFN-γ by western blot. To enrich TAPBPR levels enabling detection on western blot, the protein was immunoprecipitated using the anti-human TAPBPR antibody PeTe4. Calnexin was detected in the input as a control. HC = heavy chain. (**B-C**) Cells were treated with IFN-γ and stained for intracellular TAPBPR (B) or HLA class I (C). (**D**) Wild-type (WT) and TAPBPR^KO^ HeLa cells were transduced with the indicated HBV ORFs. Their expression levels were validated by western blot by immunoblotting for the FLAG tag. Calnexin was used as a loading control.

**Figure S7. Anchor residues of the HeLa TAPBPR^WT^ immunopeptidome are not altered by the introduction of HBV proteins.** HLA class I annotation on dataset 4 was performed using SYFPEITHI 1.0 (Schuler *et al*., 2007, Methods Mol Biol) and NetMHCpan 4.0 (Jurtz *et al*., 2017, J Immunol). Seqlogos (Tareen *et al*., 2020, Bioinformatics) were generated using all peptides (cellular and HBV) of 9 amino acids in length. Dataset 4 using HeLa TAPBPR^WT^ cells transduced with HBsAg or Pol (**A**) or HBxAg or HBcAg (**B**). HeLa TAPBPR^KO^ cells showed similar results. Peptide count used to generate each seqlogo is indicated with n. Reference seqlogos for each HLA class I allotype can be found at <http://mhcmotifatlas.org>.

**Figure S8. Gating to equalise TAPBPR levels in peptide loading assay.** Wild-type HeLaM cells (no surface TAPBPR) or HeLaM transduced with TAPBPR^WT^ or TAPBPR^Øloop^ (mutated K22-D35 editing loop (Ilca *et al*., 2018, eLife)) were treated with IFN-γ. Cells were incubated for 15min with peptide followed by staining for surface TAPBPR. (**A**) Shown is an example of the gating on TAPBPR medium-positive cells (**B)** Example of surface TAPBPR MFI after gating on to equalise TAPBPR levels. Data is shown as mean+SD of 3 technical repeats.

## 1.2 Supplementary Tables

**Table S1. HBV-derived peptides selected for validation of HLA class I binding**. ^#^ K* indicates TAMRA label on a lysine residue. ^ Anchor residues described at http://mhcmotifatlas.org are underlined. ^$^ IEDB (Vita *et al.*, 2019, Nucleic Acids Res) as of November 2024.

| **Protein** | **Peptide^#^** | **Original sequence^** | **Length** | **IEDB^$^** | **Allele** |
| --- | --- | --- | --- | --- | --- |
| Pol | SLDVSK*AFYHL | SLDVSAAFYHL | 11 | NO | A*02:01 |
| Pol | SLYK*DSPSV | SLYADSPSV | 9 | YES | A*02:01 |
| HBsAg | FLLTRIK*TI | FLLTRILTI | 9 | B*08:01 new | B*08:01 |
| HBcAg | NMK*LKIRQL | NMGLKIRQL | 9 | NO | B*08:01 |
| HBsAg | IPIPSK*WAF | IPIPSSWAF | 9 | YES | B*35:01 |
| HBsAg | ARFSWLK*LL | ARFSWLSLL | 9 | NO | C*06:02 |
| Pol | VSIPWTHK*V | VSIPWTHKV | 9 | NO | C*06:02 |

**Table S2. HLA-A*68:02-restricted HBV peptides selected for validation of the impact of TAPBPR on peptide presentation**. ^#^ K* indicates TAMRA label on a lysine residue. ^ Anchor residues described at http://mhcmotifatlas.org are underlined. ^$^ IEDB (Vita *et al.*, 2019, Nucleic Acids Res) as of November 2024.

| **Protein** | **Peptide^#^** | **Original sequence^** | **IEDB^$^** | **Allele** | **TAPBPR** |
| --- | --- | --- | --- | --- | --- |
| - | ETVSK*QSNV | ETVSEQSNV | - | A*68:02 | - |
| Pol | DVSAAFK*HL | DVSAAFYHL | NO | A*68:02 | WT & KO |
| Pol | FTFSPTYK*A | FTFSPTYKA | A*68:02 new | A*68:02 | KO |
| Pol | FTAVTNFK*L | FTSAICSVV | A*68:02 new | A*68:02 | WT & KO |
